# Acid pH Strategy Adaptation through *NRG1* in *Ustilago maydis*

**DOI:** 10.3390/jof7020091

**Published:** 2021-01-28

**Authors:** José Alejandro Sánchez-Arreguin, José Ruiz-Herrera, F. de Jesus Mares-Rodriguez, Claudia Geraldine León-Ramírez, Lino Sánchez-Segura, Patricio Adrián Zapata-Morín, Jordan Coronado-Gallegos, Elva Teresa Aréchiga-Carvajal

**Affiliations:** 1 Laboratorio de Micología y Fitopatología, Unidad de Manipulación Genética, Facultad de Ciencias Biológicas, Universidad Autónoma de Nuevo León, 66451 San Nicolás de los Garza, Nuevo León, Mexico; alejandro.sanchez@cinvestav.mx (J.A.S.-A.); fmaresr@uanl.edu.mx (F.d.J.M.-R.); patricio.zapatamor@uanl.edu.mx (P.A.Z.-M.); jordancoronadog@gmail.com (J.C.-G.); 2 Departamento de Ingeniería Genética, Unidad Irapuato, Centro de Investigación y de Estudios Avanzados del Instituto Politécnico Nacional, Km 9.6, Libramiento Norte, Carretera Irapuato-León, 36821 Irapuato, Guanajuato, Mexico; jose.ruiz@cinvestav.mx (J.R.-H.); claudia.leon@cinvestav.mx (C.G.L.-R.); lino.sanchez@cinvestav.mx (L.S.-S.)

**Keywords:** *Ustilago maydis*, *NRG1*, pH, transcript regulation, hyphae, filamentation

## Abstract

The role of the *Ustilago maydis* putative homolog of the transcriptional repressor *ScNRG1*, previously described in *Saccharomyces cerevisiae*, *Candida albicans* and Cryptococcus neoformans, was analyzed by means of its mutation. In *S. cerevisiae* this gene regulates a set of stress-responsive genes, and in *C. neoformans* it is involved in pathogenesis. It was observed that the *U. maydis* *NRG1* gene regulates several aspects of the cell response to acid pH, such as the production of mannosyl-erythritol lipids, inhibition of the expression of the siderophore cluster genes, filamentous growth, virulence and oxidative stress. A comparison of the gene expression pattern of the wild type strain versus the *nrg1* mutant strain of the fungus, through RNA Seq analyses, showed that this transcriptional factor alters the expression of 368 genes when growing at acid pH (205 up-regulated, 163 down-regulated). The most relevant genes affected by *NRG1* were those previously reported as the key ones for particular cellular stress responses, such as *HOG1* for osmotic stress and *RIM101* for alkaline pH. Four of the seven genes included *WCO1* codifying PAS domain ( These has been shown as the key structural motif involved in protein-protein interactions of the circadian clock, and it is also a common motif found in signaling proteins, where it functions as a signaling sensor) domains sensors of blue light, two of the three previously reported to encode opsins, one vacuolar and non-pH-responsive, and another one whose role in the acid pH response was already known. It appears that all these light-reactive cell components are possibly involved in membrane potential equilibrium and as virulence sensors. Among previously described specific functions of this transcriptional regulator, it was found to be involved in glucose repression, metabolic adaptation to adverse conditions, cellular transport, cell rescue, defense and interaction with an acidic pH environment.

## 1. Introduction

Microbial pathogens undergo remarkable cellular alterations to adapt to changes in the environment. Nutrient deprivation, sudden pH changes, oxidative stress and certain toxic compounds activate this adaptive response. Low or slow ability to adapt to external environmental variations incapacitate cells to function correctly in non-optimal growth conditions. Fungi, in particular, stand out by how they confront environmental pH changes, firstly through an efficient change in the expression of a wide range of genes, and secondly through adjusting physiological and morphological features. Eventually, cell shape determination involves integrating an entire pattern of signals from many cellular processes [1,2,3].

*S. cerevisiae* possesses two *NRG* genes: *NRG1* and *NRG2*. These genes regulate a set of stress-responsive genes whose mutation decreases the cells’ freezing tolerance and enhances resistance to salt and oxidative stress [4]. The *NRG1* gene encodes a 25-kDa C2H2 zinc finger protein that specifically binds to two consensus regions located upstream of the glucoamylase activation sequence (glucan 1, 4-alpha-glucosidase) *STA1* gene. Disruption of the *NRG1* gene causes a five-fold increase in the *STA1* gene transcript level in the presence of glucose in *S. cerevisiae* [5]. On the other hand, in *C. albicans*, the only *NRG1* gene codifies for a DNA-binding transcriptional repressor of the filament-specific genes *HYR1, ALS8, HWP1* and *ECE1*, under non-filament-inducing conditions. A decrease in the *NRG1*p gene transcript induces filamentous growth [6,7]. It has also been shown that *NRG1* is down-regulated during hyphal initiation by two distinct mechanisms: in *C. albicans*, *NRG1* expression is inhibited by the cAMP-PKA pathway, whereas release from farnesol inhibition (a quorum-sensing molecule) during inoculation activates Nrg1p degradation [8,9,10,11]. In other scenarios, Nrg1 begins to disappear within minutes upon serum induction at 37 °C in wild-type cells, by its transcription repression and degradation [11]. Under certain filament-inducing conditions, *NRG1* transcripts are down-regulated, allowing the expression of filament-specific genes. Moreover, Nrg1 is transiently displaced from hypha-specific promoters via the activation of the cAMP-PKA pathway growing in YPD (Yeast extract, Peptone, Dextrose) solid medium with 10% serum at 37 °C [10]. In *Cryptococcus neoformans*, *NRG1* controls the induction of genes involved in virulence, mating and cell wall integrity along with those for carbohydrate metabolism and oxidative stress response proteins, including the *UGD1* gene, which encodes a UDP-glucose dehydrogenase needed for polysaccharide capsule production [5,12].

Basidiomycota, like other multicellular eukaryotes, have evolved systems to overcome adverse environmental conditions and the biotrophic fungus *U. maydis* is no exception [13]. When growth conditions, such as pH, change, expression of genes involved in general metabolism, cellular cycle, transport and cellular rescue, are adjusted in order to improve survival possibilities [3,14]. Functional categories of genes whose expression patterns change during transition from neutrality to pH 3 have been described before [3], as well as the in vitro yeast-to-mycelium dimorphic switch that occurs when a change in the pH of the growing medium takes place [15]. This process is similar to the one that occurs with several human pathogenic fungi [16]. It is well known that the signaling pathways cAMP-dependent protein kinase (PKA) and the mitogen-activated protein (MAP) kinase regulate the pH-dependent dimorphism in *U. maydis* [17]. The presence and function of the putative *HOG1* homolog gene (UMAG_02357) in *U. maydis,* however, has not yet been characterized (at least not in vitro or in vivo); on the other hand, it has been reported that the transcription factors Hog1 and Rim101 in *C. albicans*, and the homolog of PacC in *Aspergillus nidulans* are involved in the yeast-to-hypha switch at low pH [18]. The RIM101/pacC transcription factor has been recognized to control the pH-response in several fungal species, but not in the dimorphism and pathogenesis of *U. maydis* [19]. Because of this, we analyzed promoter sequences of the Rim101 genes regulated at acid pH (in preparation) and observed that *RIM101* has motifs in its promoter region that could be recognized by *NRG1*. To describe whether the transcriptional repressor *NRG1* was involved in the process of acid pH adaptation, we have proceeded to the identification and deletion of the only *NRG1* homolog (predicted by structure similarity) gene in *U. maydis*. The data obtained show that the *NRG1* gene product behaves as a transcription factor up-regulating 205 genes and repressing 163 at acid pH. Here, we have analyzed the role of *NRG1* in different aspects of the metabolism and behavior of *U. maydis*.

## 2. Material and Methods

### 2.1. Strains, Media and Culture Conditions

*U. maydis* strains used in this study were FB2 [20], BMA2 (*a2b2* ∆*rim101*) [19] and FMA2 (*a2b2* ∆*nrg-1*), generated in this study (Table 1). *U. maydis* strains were maintained in 50% glycerol at −70 °C. Cells were recovered in liquid CM medium [21] and incubated for 48 h at 28 °C under shaking conditions, to be used as inoculum for other cultures. When necessary, the culture media were supplemented with carboxin (10 µg/mL). Growth of *U. maydis* in liquid CM or minimal medium (MM) [21] was measured by its optical density (OD) at 600 nm. *Escherichia coli* DH5α was used for plasmid DNA cloning; the plasmid used was *pDonr*Cbx122, containing the *U. maydis* carboxin resistance gene [22].

### 2.2. Oxidative Stress

To test the sensitivity to oxidative stress by H_2_O_2_, 10^6^ cells mL^−1^ of each strain were distributed on CM agar plates, and circles of filter paper of 0.5 cm in diameter soaked with 0.8 M H_2_O_2_ were placed on top of the medium and incubated at 28 °C for 48 h. Halos of growth inhibition were measured on each paper circle.

### 2.3. Identification of NRG1 Gene of U. maydis

Reported sequences of Nrg1p from other fungi were used to generate an amino acid consensus sequence. This consensus sequence was used to identify a region in the *U. maydis* genome where the *NRG1* gene is located. Once an open reading frame had been located within the probe’s alignment region, a BLAST was performed against the NCBI (Bethesda, MD, USA) database to confirm that this ORF aligns with sequences already reported for Nrg1p in other fungi.

### 2.4. Determination of Virulence

Pathogenicity assays were performed as previously described [23]. Accordingly, *U. maydis* cultures of complementary strains were separately grown overnight at 28 °C. Cells were recovered by centrifugation, washed with sterile distilled water, and suspended in sterile distilled water to a density of 10^8^ cell/mL, and equal volumes of the suspensions were mixed. Aliquots of 0.2 mL of the resulting mixture were inoculated with syringe and needle into the second node of 7-day-old maize seedlings cv. Cacahuazintle. Plants were grown in a greenhouse under regulated conditions, and symptoms were observed after 7 to 14 days.

### 2.5. DNA Extraction

Genomic DNA from *U. maydis* was isolated using the glass bead lysis method [24]. Its absorbance was measured at 260 nm with a Nanodrop (Thermo Fhisher Scientific; Santa Clara, CA, USA), and its integrity observed by electrophoresis in agarose gels (Sigma-Aldrich; St. Louis, MO, USA).

### 2.6. Mutation of U. maydis NRG1 Gene

*NRG1* mutants were constructed by the double joint PCR method [25]. Primers designed to construct the deletion cassettes of the *NRG1* gene are shown in Supplementary Table S1. PCR reactions were conducted using High Fidelity Platinum *Taq* DNA polymerase (Invitrogen, Carlsbad, CA, USA), using the following general program: an initial step of 94 °C for 5 min; amplification (35 cycles) at 94 °C for 30 s, followed by annealing at a primer-specific temperature for 30 s, polymerization at 68 °C (1 min/kb of DNA target length) and finally an extension period of 7 min at 68 °C. For the first PCR reaction, 100 ng of genomic DNA template were used to amplify the 5′ and 3′ flanking regions of *NRG1* (primers NRG1-A, NRG1-BCbx, NRG1-CCbx and NRG1-D). In a parallel PCR, 5 ng of *pDonr*Cbx [22] were used for Cbx marker amplification. In the third step PCR, deletion cassettes were amplified using 1 µL of the purified second PCR products as a template, and NRG1-E and NRG1-F primers (Their sequence are shown in Supplementary Table S1). Once the correct fragment size and restriction pattern of the deletion cassette was confirmed, it was purified using Pure Link Quick PCR purification columns (Invitrogen, Carlsbad, CA, USA), and 5 µg of purified DNA was used for PEG-mediated protoplast transformation of the wild type strain FB2 (*a2b2*) as previously described [26]. The transformed protoplasts were spread over DCMS double-strength agar plates of selective medium containing 1 M sorbitol (Sigma-Aldrich; St. Louis, MO, USA), and incubated at 28 °C for five days. After two rounds of sub-cultivation in liquid selection medium containing Carboxin (10 µM), DNA was isolated from transformants using standard protocols, and the disruption event and proper location were confirmed by PCR.

### 2.7. Analysis of the Phenotypic Characteristics of nrg1 Mutant Strains of U. maydis

Strains were grown under standard conditions in liquid CM. Cells were pelleted by centrifugation, washed twice, and suspended in distilled water to a density of 10^8^ cells/mL. Ten-fold serial dilutions of the cell suspensions were made, and 10 µL aliquots from 10^3^ to 10^8^ dilutions were spotted on plates of solid MM adjusted to pH 7 and supplemented with each of one of the selected stressor agents. Plates were then incubated at 28 °C for 48–72 h and photographed. Growth rate was measured in MM pH 7 or MM pH 4 supplemented with 1% of glucose (Sigma-Aldrich; St. Louis, MO, USA). Cells were inoculated to a density of 0.005 OD600 units, and the cultures were incubated under shaking conditions at 28 °C and 180 rpm. Samples were taken every 6 h through a total of 72 h, and the OD at 600 nm was measured. These experiments were conducted three times. Cells shape and their characteristics were observed with a multiphoton microscopy system (LSM 880-NLO, Zeiss, Germany; Waltham, MA, USA). For some experiments, cells were stained with a solution containing solophenyl flavine 0.1 % (Sigma-Aldrich; St. Louis, MO, USA) and propidium iodide 0.002% (Sigma-Aldrich; St. Louis, MO, USA), thoroughly washed by centrifugation, and observed by epifluorescence. To determine cell size, 100 cells of each strain were randomly selected and measured with ZEN 3.2 blue edition (LSM 880-NLO, Zeiss, Germany; Walthman, MA, USA) software. For yeast and hyphae count, strains were grown for 72 h in liquid pH 4 MM. Samples were recovered after 24, 48 and 72 h of incubation, and stained with calcofluor white (Sigma-Aldrich; St. Louis, MO, USA). Three hundred cells were counted for each strain. Statistical analyses were performed by one-way ANOVA and Tukey’s b test in IBM SPSS v25.0 (IBM Corp, Armonk, NY, USA, and graphs were made in OriginPro 2020b (OriginLab Corporation; Northampton, MA, USA) data and graphing software (*p* < 0.05).

### 2.8. RNA Extraction

After 14 h of growth, cells were recovered from MM pH 4 by centrifugation, and total RNA was extracted from frozen and ground fungal tissue using the PureLink RNA Mini Kit (Invitrogen, Carlsbad, CA, USA) method according to the manufacture’s protocol. RNA integrity was evaluated by formaldehyde-agarose gel electrophoresis [27].

### 2.9. RNA-Seq Library Sequencing

Synthesis of cDNA libraries for RNAseq was made according to the TruSeq RNA sample prep v2 protocol (Illumina). Each library was sequenced using NextSeq 500 platform in paired-end mode with a read length of 2 × 150-bp. The RNAseq data discussed in this publication have been deposited in NCBI’s Gene Expression Omnibus [28] and are accessible through GEO Series accession number GSE133840 (https://www.ncbi.nlm.nih.gov/geo/query/acc.cgi?acc=GSE133840).

### 2.10. Analysis of the RNA-Seq Data

Libraries of forward and reverse raw samples were obtained from the Illumina sequencing.

RNA-seq data analysis was done by combining Bioconductor [29] and BLAST2GO (version 5.1) outputs [30]. Quality control and alignment read with a reference genome section were made with Bioconductor, and the differential gene expression (DGE) and GSEA enrichment analysis on BLAST2GO.

Quality-control checkpoints. The raw reads were processed by Bioconductor QuasR utility preprocess reads. The chosen parameters to filter were as follows: Quality < Q20, minimum lengths of 130 nt (after adapter removal), trimmed read lengths of 130 nt (to remove low-quality sequences). Additionally, GC content, over-represented k-mers and duplicate reads were taken into consideration.

Read alignment. Reads were mapped to the reference genome of *U. maydis* 521, GenBank assembly accession: GCA_000328475.2, submitted by the Broad Institution 06-50-2017. Using QuasR [31,32,33]. Thus, we obtained the Binary Alignment Map (BAM) files for each sample. Gene transcript expression is based on the number of reads that map to each transcript sequence from the gene transfer format (GTF) file of *U. maydis* 521.

### 2.11. Differential Gene Expression (DGE) and GSEA Analysis

BAM files were employed to generate the count tables that BLAST2GO needed to run the DGE and GSEA routines. Differentially expressed genes were those that showed at least a 2.0-fold change in expression. All differentially expressed genes were classified into the categories of molecular function, cellular component and biological process in the Gene Ontology (GO) database (http://www.geneontology.org/). FB2 samples were considered basal and FMA luminal; default BLAST2GO parameters were used, and only GO terms with *p* < 0.05 were included in the present study. Gene Set Enrichment Analysis (GSEA) was used to find enriched GO terms, *p*-value *p* < 0.05. Additionally, an enrichment analysis was performed by STRING, a functional protein association network, available at https://string-db.org/, for up- and down-regulated genes interaction network and functional domains analysis [34]. Finally, the protein location was determined by Gene Ontology (GO) in the Uniprot database (https://www.uniprot.org).

### 2.12. Validation of Expression (RT-qPCR)

Validation of the expression profile was developed by quantitative reverse transcription PCR (RT-qPCR) for several aleatory selected genes. Total RNA was isolated from the strains described above, treated with RNase-free DNase, and cDNA was synthesized using oligo (dT) primers from the SuperScript first-strand synthesis reverse transcription kit (Invitrogen, Carlsbad, CA, USA). According to the manufacture’s specifications, cDNA was used as a qPCR template using the KAPA SYBR green quantitative PCR master mix (Kapa Biosystems, Wilmington, MA, USA). The Step One Real-Time PCR system (Applied Biosystems, Foster, CA, USA) was used as the fluorescence detector with the following PCR conditions: an initial denaturing cycle of 95 °C for 3 min and 40 cycles of denaturation at 95 °C for 10 s and annealing/extension at 53 to 93 °C with fluorescence monitoring each 0.5 °C. These data confirmed a single product amplification for each primer pair and the lack of primer dimerization. Gene amplification for each strain and condition were made with the actin gene behaving as constitutive in all conditions to normalize all data [35]. The primers used are described in Supplementary Table S2.

### 2.13. Statistics

All data were processed using R Core Team (https://www.r-project.org/) [36]. We used Shapiro-Wilk to first explore the distribution form of the data. If the data satisfied standard distribution patterns, a parametric test was used for statistical analysis, and we applied one-way ANOVA tests to analyze the differences among those groups. If the data were not normally distributed, non-parametric tests were carried out. We used the Kruskal-Wallis test (commonly used for several independent samples in non-parametric assays) to analyze differences among the groups. When the data were parametric, we used Tukey’s test.

### 2.14. Promoter Analyses

We selected the promoters for the differential genes from the 2-Kb upstream sequence, using the NCBI database for *U. maydis*. Whole sequence was analyzed in the discoverer software [37] to find consensus motifs recognized for transcription factors from the Yeastract database [38]. Promoter genes with the *NRG1*, *RIM101*, *YAP1* and *HOG1* sites were selected along with their position and repetition.

### 2.15. Glycolipids Detection

*U. maydis* cells of FMA2, BMA2 and FB2 strains were grown 96 h at 28 °C in liquid YEPS (1% yeast extract, 2% peptone and 2% sucrose) to the exponential phase, and then shifted to fresh YNB-N with 1.7 g/L yeast nitrogen base without ammonium sulfate and 5% glucose as carbon source (BD; Franklin Lakes, NJ, USA). MEL’s production was carried out as previously described [39], using wild type cells as control under nitrogen starvation. Samples were harvested at 24 h, 48 h, 72 h and 96 h of incubation, and treated with ethyl acetate [1:1] (Sigma-Aldrich; St. Louis, MO, USA). Ethyl acetate was evaporated, and the glycolipids were suspended in methanol. Samples of 5 µL were placed on a silica gel 60 F254 thin-layer chromatography (TLC) column (Sigma-Aldrich; St. Louis, MO, USA). Chromatography was performed with an eluent of chloroform-acetone-water (30:60:2). The silica gel column was dried at room temperature and visualized after exposure to 0.1 N KMnO_4_ in 0.05N H_2_SO_4_ (Sigma-Aldrich; St. Louis, MO, USA).

### 2.16. Nucleus and Cell Wall Staining

*U. maydis* cells were fixed by addition of 4% paraformaldehyde (Electron Microscopy Science; Hatfield, PA, USA) dissolved in 0.16 M phosphate buffer solution pH 7.4 and incubated overnight; samples were washed three times with phosphate buffer and stored at 4 °C. Cells were sedimented by centrifugation (3650 g, 10 min), suspended in 100 µL deionized water and 40 µL of 0.002% propidium iodide (Sigma Aldrich, St. Louis, MO, USA) dissolved in deionized water, and samples were incubated during 15 min in darkness. Another round of centrifugation (3650 g, 10 min) was done, cells were suspended in 100 µL deionized water, and 2 µL of 0.1% solophenylflavine 7GFE or direct yellow 96 (Sigma-Aldrich; St. Louis, MO, USA) dissolved in deionized water, added and incubated for 10 min in darkness. Three times washed with deionized water, and 2 µL were placed over a transparent adhesive membrane (ed Pella Inc. Redding, CA, USA) mounted on glass slides, covered with high-performance cover glass slide (D = 0.17 mm ± 0.005 mm refractive index = 1.5255 ± 0.0015, Abbe number = 56 ± 2) and observed in the multiphoton microscope system (LSM 880-NLO, Zeiss, Germany; Walthman, MA, USA). In all experiments, microscope operating conditions were lasers at 458 nm and 543 nm with 1.0% and 2.2% of power, respectively. Observations were performed with an oil immersion objective 63X/1.40 DIC, NA ∞−0.17, Zeiss Plan NEOFLUAR. Images were acquired by separation of the emission into two channels, green region for solophenyl flavine stain (481–543 nm) and red region for propidium iodide stain (605–670 nm). All micrographs were captured in CZI format at 2048 × 2048 pixels and RGB.

## 3. Results

### 3.1. In Silico Identification of the Gene NRG1 Based on Sequence Similarity

**Figure 1:**
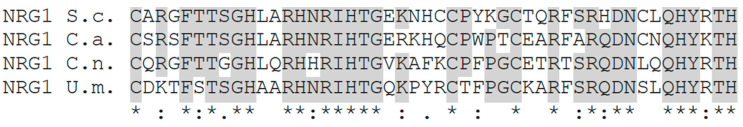
Amino acid sequence alignment of the conserved domain of NRG1. Identically conserved residues are shaded, asterisks mark entirely conserved residues and asterisk and dots indicate similar residues. (S. c: *Saccharomyces* *cerevisiae*, C. a: *Candida* *albicans*, C. n: *Cryptococcus* *neoformans*, U. m: *U. maydis*).

In previous studies we found a very high percentage of NRG-binding sites in the DNA promoter’s regions of pH-responsive genes in the Basidiomycota *U. maydis* (unpublished data derived from [14]). Because *NRG1* has been described as a transcriptional repressor [4,7], we hypothesized that the Nrg1 homologous protein might be involved in regulating the pH response besides or in conjunction with RIM101/PacC in this fungus. Thus, we designed a strategy to track down this gene with an *in silico* molecular probe in the *U. maydis* genome. A single copy predicted protein encoded by this putative ORF Umnrg1p (UMAG_15036) is slightly similar to their *C. albicans* and *S. cerevisiae* counterparts (Figure 1). The identified gene lacks introns and encodes a protein of 622 amino acids that shows 22.31% identity to *C. albicans* and 23.96% identity to *S. cerevisiae* Nrg1. The identity region is located mainly at the functional zinc finger domain, suggesting that functions as a transcription factor. Additionally, the *U. maydis* Nrg1 protein is about twice the size compared to its counterparts. Analysis of the regulatory region revealed that it contains four putative in silico predicted binding sites that are responsive to pH through Rim101 in its promoter region.

### 3.2. Mutation and Phenotypic Characterization of the nrg1 Mutant Growth and Wall State under Different Conditions

**Figure 2:**
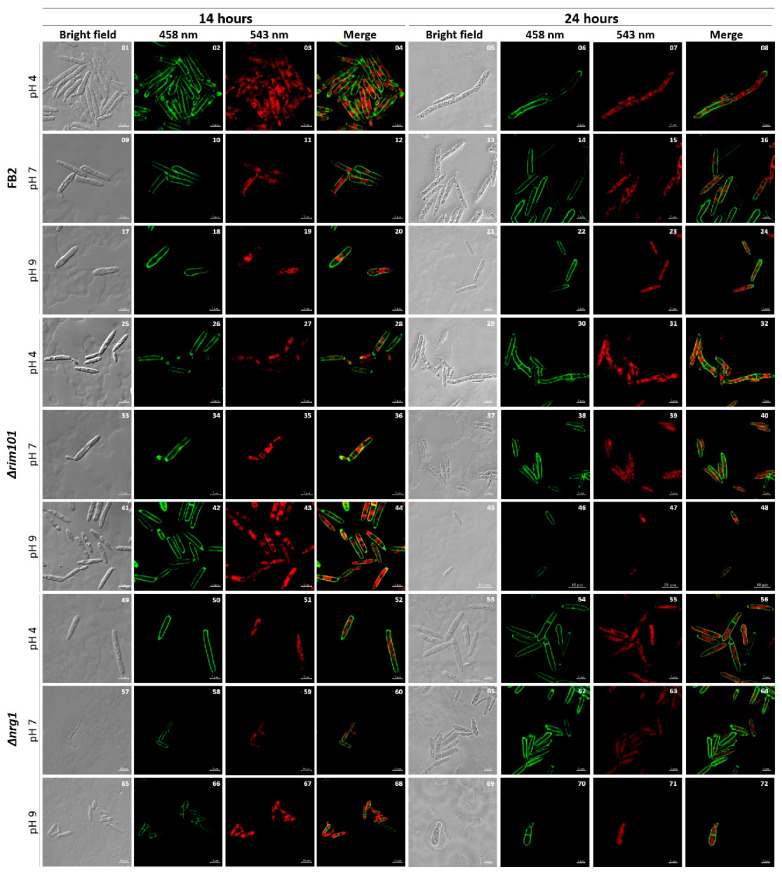
FB2, Δ*rim101* and Δ*nrg1* strains cell morphology. Cells of wild type (control), BMA2 and FMA2 were grown at pH 4, pH 7 and pH 9. After 14 h and 24 h of growth, they were photographed. Cells were stained with propidium iodide chromosomal content and with solophenylflavine to detect fungal cell walls and septum. The scale bar represents 10 µm. The experiment was done in triplicate.

To achieve an insight of *NRG1* functional gene product in *U. maydis*, we replaced it by means of homologous recombination using the carboxin resistance cassette [22]. One of the obtained Δ*nrg1* mutants (FMA2) was further characterized. Firstly, the growth behavior and morphology of FMA2 (*nrg1*), BMA2 (*rim101*) and FB2 (wild type) in liquid MM at pH 4, pH 7 and pH 9 were compared. The results obtained (Figure 2) show that, as reported [19], the growth rate of the *rim101* mutant cells is similar to the one of the wild type strain, except for the lower amounts of hyphae formed at pH 4 compared to the wild type, and the reduced cell size at pH 9. Similarly, the *nrg1* mutant showed less hypha formation at pH 4 than the wild type, and at pH 9 also showed a smaller cell size. Statistical analysis of cell size showed that at pH 4 and 14 h of growth, the *nrg1* mutant did not show significant differences in size compared to the wild-type FB2 strain, but under the remaining conditions, the *nrg1* mutant showed significant differences in cell growth compared to FB2, but their cell size was smaller under all treatments (Supplementary Figure S1). Another remarkable characteristic was the differential frequencies of septum deposition. As shown in Figure 2, after 24 h of growth at pH 4, the number of septa increased in the cells of mutants *nrg1* and *rim101*, compared with wild type strain cells. Morphological changes among unicellular and filamentous forms are crucial to fungal pathogen’s virulence.

**Figure 3:**
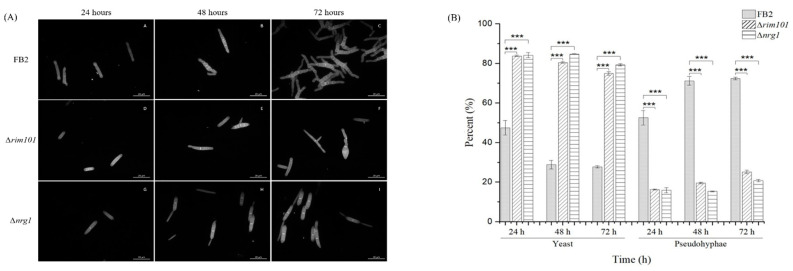
Growth of FB2, Δ*rim101* and Δ*nrg1* strains on MM (minimal liquid medium) pH 4. (**A**) Cells of WT, Δ*rim101* and ∆*nrg1*; strains were grown in MM pH 4 and analyzed for 72 h. (**B**) Yeast and pseudohyphae proportion, *U. maydis* wild-type (dotted bar), rim101 mutant (diagonal filled bar) and nrg1 mutant (vertical line bar) grown on MM (minimal liquid medium) pH 4. Bars represent the percentage of yeast and pseudohyphae at 24, 48 and 72 h. The growth morphology of each mutant strain was compared with the wild-type strain. The bar graph represents the mean ± SEM; *** *p* < 0.001. Tukey’s test was performed, and reliability used was 95%. Scale bar: 10 µm.

The phytopathogen *U. maydis* forms a hypha that penetrates their host at the early stages of infection [13]. In vitro, the phenotypic transition of this Basidiomycota can be triggered by the pH change in growth medium [15]. It also has been reported that *NRG1* in *C. albicans* affects filamentous growth [7]. To explore the possibility of a similar behavior in *U. maydis, rim101* and *nrg1* mutants were grown at different pH values. Wild-type *U. maydis* grows yeast-like in MM at pH 7, and in mycelial form at acid pH [15]. However, in MM at pH 4 with a buffer to maintain acid pH to induce hyphal growth, a general delay in mycelium formation in both mutants was observed. For example, after 24 h of growth, only 50% of the wild type cells remained in yeast form, compared to 84% of the BMA2 and FMA2 cells and, after 48 h of growth, only 29% of the wild type cells were still in the yeast form. In comparison, in the *rim101* and *nrg1* mutants, the percentage of yeast cells was 80% after 72 h of growth. At pH 4, 27% of the wild type cells were still in the yeast form, whereas the values for the *rim101* and *nrg1* mutants were, respectively, 74% and 79%. Since *rim101* and *nrg1* mutants displayed similar morphological phenotypes, it may be suggested that both *RIM101* and *NRG1* could be participating in the development of mycelium at acid pH. Since *NRG1* in *U. maydis* is probably regulated by the transcriptional factor Rim101, these data suggest that Nrg1 could be the main contributor involved in this developmental process (Figure 3). Accordingly, evidence exists that the *NRG1* gene product plays a role in related phenotypic characteristics, even if filamentation triggering signals differences in *C. albicans* and *U. maydis* are considered. As for phenotype and gene expression under these conditions, the enrichment analysis showed a direct participation of a Cdc24p *S. cerevisiae* probable homolog that is necessary for maintaining cell polarity [40,41]. This homolog appears to be encoded by the UMAG_02244 gene whose calponin domain or CH is present in signaling proteins involved in the regulation of smooth muscle contraction [42,43,44]. Its presence in *C. albicans* is required for hyphal growth in response to serum-containing medium [45]. Furthermore, its serine-threonine protein kinase domain makes this protein more interesting because, according to STRING database, it interacts with elements of the PKA and MAPK pathways, and a *Ras-GTPase* gene; all these key elements are involved in the maintenance of hyphal growth [45].

### 3.3. Altered Resistance of ∆nrg1 Mutant Cells to Oxidative, Toxic Ionic and General Stress

**Figure 4:**
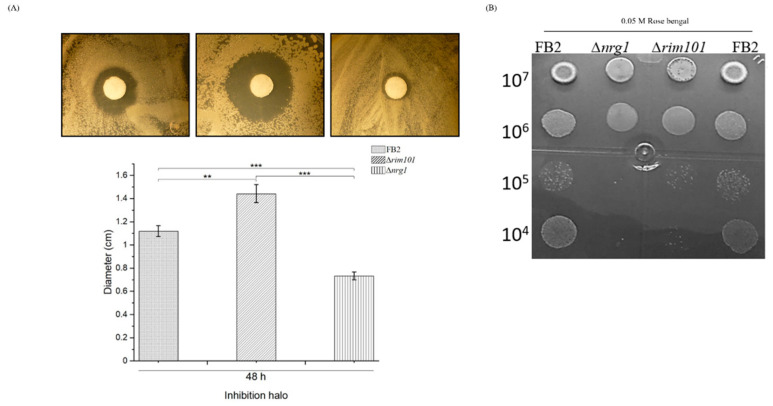
FB2, Δ*rim101* and Δ*nrg1* mutant strains effect of oxidative stress. (**A**) Wild type FB2 (*a2b2*) and Δ*nrg1* mutant derived from this strain was subjected to H_2_O_2_ treatment as described in the text. (**B**) Wild type, ∆*rim101* and ∆*nrg1* strains were grown on Rose Bengal 0.05 M, for 48 h at 28 °C. Results of a total of three different experiments using three plates per experiment are represented. Bar graph represents the mean ± SEM; ** *p* < 0.005, *** *p* < 0.001. Tukey’s test was performed, and reliability was 95%.

In *S. cerevisiae*, several genes responsive to oxidative stress are regulated by the transcriptional activator Yap1. Nrg1/Nrg2 regulates 14 genes (*SGA1*, *GSY1*, *RP11*, *SHC1*, *PRY3*, *NDH1*, *FRE4*, *GAL7*, *ELO1*, *ICY1*, *IDH2*, *RSB1*, *YH033W* and *YGR050C*) in cells exposed to hydrogen peroxide [4,46]. In order to evaluate the physiological importance of *NRG1* in stress responses, we tested *rim101* and *nrg1* mutant cells of *U. maydis* to oxidative stress. Cells were spread on a plate and exposed to H_2_O_2_ contained in paper disks as explained above. The oxidative reagent caused a larger zone of growth inhibition for the *rim101* mutant, followed by the wild-type cells; strikingly, in the *nrg1* mutant strain, the halo of growth inhibition was smaller (Figure 4A). This could be an indication that the responsible regulators of oxidative stress genes are turned on in the *nrg1* mutant strain. On the other hand, determination of the effect of Rose Bengal on the *rim101* and *nrg1* mutant strains’ growth revealed that they were more sensible than the wild type strain (Figure 4B).

### 3.4. Mannosyl Erythritol Lipids (MELs) Synthesis Pathway and Light Voltage Response Genes Are Affected in the ∆nrg1 Mutant Strain

**Figure 5:**
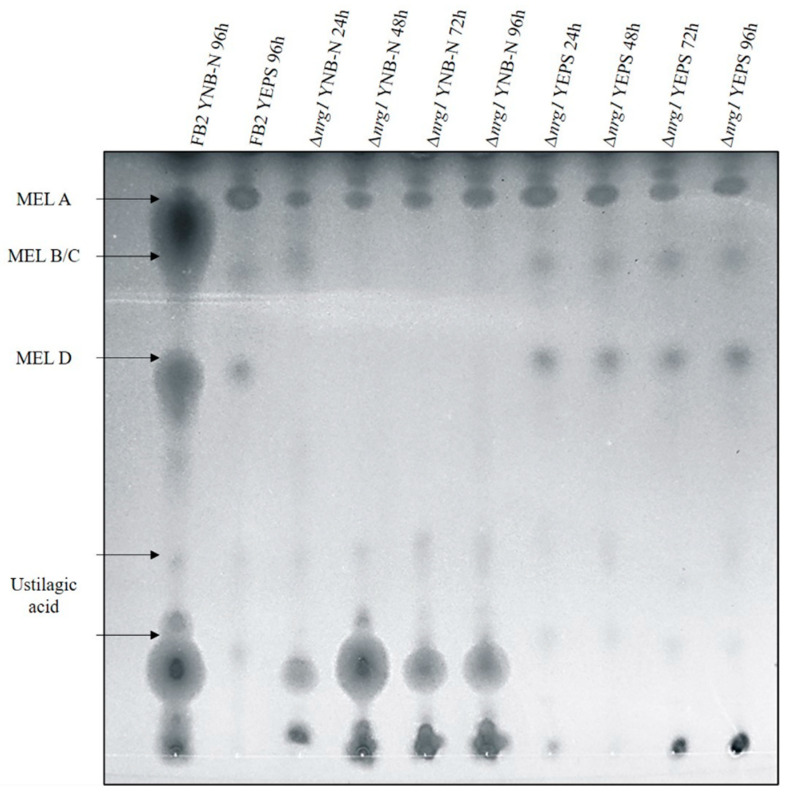
Glycolipids thin-layer chromatography (TLC) production analysis. Wild type and nrg1 mutant strains ethyl acetate extracellular glycolipid extracts were separated by TLC. MELs separate according to their degree of acetylation. Wild type strains had expected MELs charges and ustilagic acid in the specific medium. Δ*nrg1* produced slightly higher amounts than wild-type in the preculture medium and barely detectable amounts in the MELs inductive medium; moreover, ustilagic acid production also decreased.

The existence of a gene cluster encoding genes for the biosynthesis of mannosyl-erythritol lipids in *U. maydis* was reported by Hewald [47]. Since the cluster’s expression level was altered in the *nrg1* transcriptome analysis (see above), the Δ*nrg1* mutant was tested for glycolipids production under limited nitrogen conditions. Glycolipids were analyzed at different times of incubation of the Δ*nrg1* mutant and the wild type strain. It was observed that FB2 wild-type cells secreted average amounts of the MEL A, MEL B/C, MEL D and ustilagic acid in the media without a nitrogen source. On the other hand, the *nrg1* mutant cells secreted slightly larger amounts of MELs in preculture medium. Nevertheless, when incubated in the specific medium to produce MELs, there was a lower production of ustilagic acid, and the MELs production, except for MEL A, was also inhibited (Figure 5). When expression analysis was related to these findings, we found that Mac1 (UMAG_03116) was increased six-fold, and Mac2 (UMAG_10636) two-fold; both genes encode acyltransferases. Additionally, the major facilitator involved in MEL transport Mmf1 (UMAG_03115) was increased five-fold, and seven-fold in Emt1 (UMAG_03117), encoding the erythritol-mannosyl-transferase responsible of MELs production.

### 3.5. Cellular Functions of U. maydis NRG1-Regulated Genes

**Figure 6:**
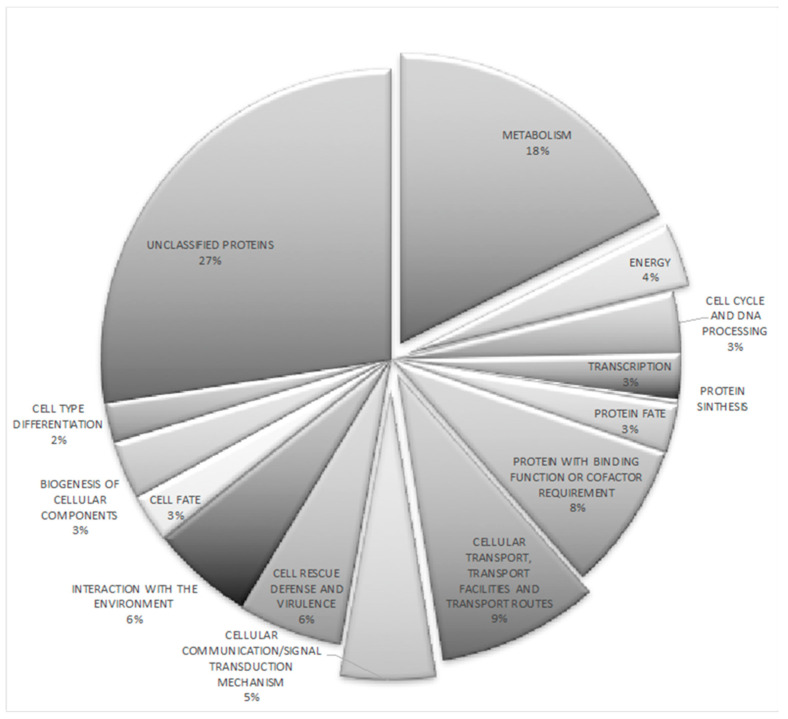
Functional categorization of the *U. maydis* 368 differentially regulated genes at pH 4 in the Δ*nrg1* mutant strain. The figure shows the percentages of differentially regulated genes corresponding to each given functional category. http://mips.gsf.de/funcatDB/.

Our next objective was to examine the cellular roles of the altered gene expression triggered by *NRG1* mutation. Thus, RNA_seq analysis of the wild type and the *nrg1* strains under acid pH conditions were carried out. Around 30 million reads were analyzed for each library, and filtrate reads were mapped on the reference *U. maydis* genome [13]. Our results indicate both the activation and repression functions of *NRG1* at acid pH. In total, comparing ∆*nrg1* mutant gene expression patterns with the wild type ones, we identified 368 differentially regulated genes; 205 genes were up-regulated and 163 were down-regulated (Supplementary Table S3). Functions of only a small proportion of *U. maydis NRG1*-regulated genes have been assessed experimentally; 27% of them encode putative proteins. On the other hand, other gene functions might be deduced on their sequence similarity to other fungal genomes. Data were grouped through the Munich Information Center for Protein Sequences (MIPS) and Functional Catalog database to classified biological functions [13,48]. Functional grouping of whole differentially expressed genes indicated that unclassified proteins (27%) and metabolism (18%) were the functional categories with the most significant number of differentially expressed genes. Other well-represented functional categories directly involved in this phenomenon were the following: (i) cellular transport with 9% of differentially expressed genes (DEGs), (ii) cell rescue defense and virulence, 6%, (iii) interaction with the environment, 6%, and (iv) cellular communication/signal transduction mechanism, 5% (Figure 6). All these functional categories agree with the reported effect of acid pH growth on the fungus [3].

### 3.6. Validation of the RNA-Seq Data by RT-qPCR

In order to validate the results from *U. maydis* transcriptome data, we selected some differentially regulated genes and analyzed their expression by real-time quantitative PCR (RT-qPCR), using the primers described in the Supplementary Table S2. The expression of each gene was calculated at pH 4 and after 14 h of growth, using the 2^−ΔΔCT^ method [49]. In general, the estimated expression was consistent with those obtained by high-throughput RNA sequencing, thus validating the RNA-Seq data (results presented in Supplementary Table S4).

### 3.7. Functional Enrichment and Network Analysis of Differentially Expressed Genes

**Figure 7:**
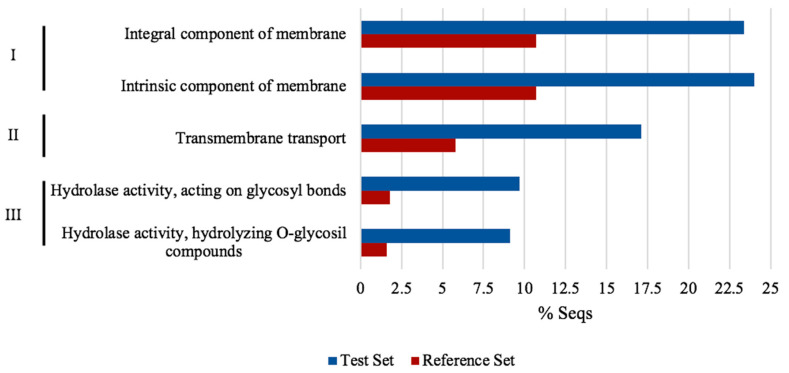
Differentially regulated genes gene ontology enrichment analysis of Δ*nrg1* strain. Graph shows enriched functional categories using whole differentially expressed genes (DEGs) by NRG1 in BLAST2GO with FDR ≤ 0.05. I, Cellular component; II, Biological process; III, Molecular function. The reference set were predicted genes in *U. maydis* genome and the test set were differential expressed genes obtained from Δ*nrg1* transcriptome at pH 4.

Functional annotation enrichment analysis was conducted to explore the relationship between the differentially expressed genes (DEGs). We used the 368 DEGs into software BLAST2GO [30], which showed 5 GO terms enriched with FDR 0.05 and a *p*-value < 0.05. The enriched GO terms were integral components of the membrane (GO:0016021), an intrinsic component of the membrane (GO:0031224), transmembrane transport (GO:0055085), hydrolase activity acting on glycosyl bonds (GO:0016798) and hydrolase activity hydrolyzing O-glycosyl compounds (GO: 0004553). Additionally, enrichment was reinforced with the string bioinformatic tool, and both results were interpreted (see Figure 7). Direct and comprehensive results from BLAS2GO and STRING transcriptome enrichment are presented in Supplementary Table S5 as cell tactics to counteract all the acid pH effects, trough gene expression/repression by *NRG1*.

### 3.8. The NRG1 Gene Is Required for U. maydis Pathogenesis

**Figure 8:**
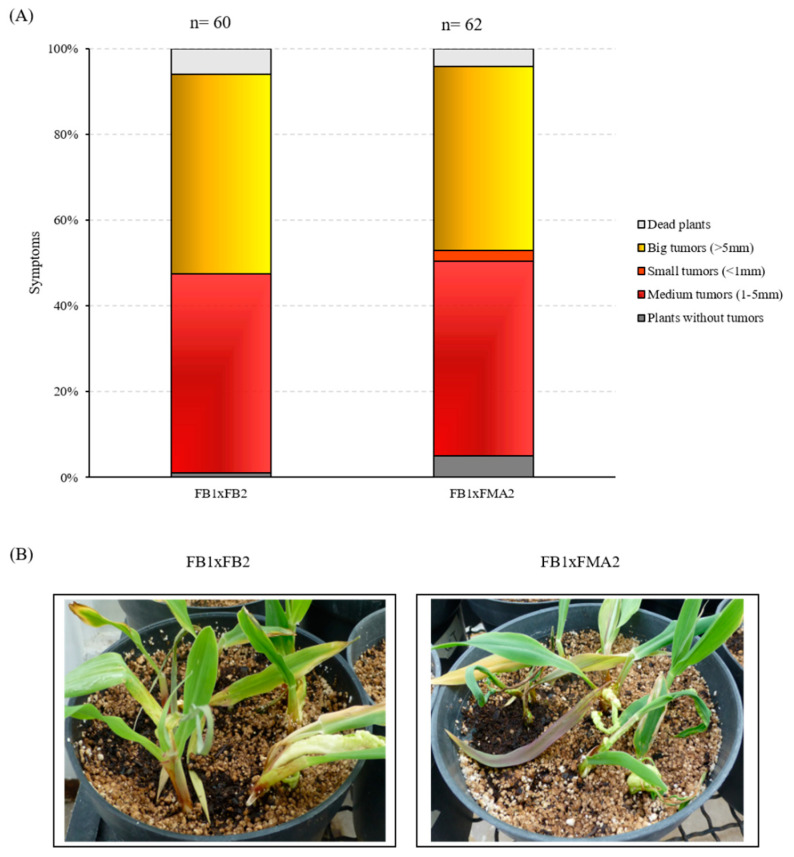
Pathogenicity assay of the *U. maydis* nrg1 mutant. (**A**) Disease symptoms were quantified based on three biological replicates with the indicated strains 14 days post-infection (dpi). The total number of infected plants is indicated above each column. (**B**) Representative images of plants infected with mixture wild-type FB1xFB2 strains or FB1xFMA2 (Δ*nrg1* mutant) strains 14 dpi. The number of infected plants is indicated at the top of the bar.

Although multiple transformation events were performed, it was not possible to obtain the mating compatible mutant strain (FB1). Accordingly, we decided to carry out this approach with the mutant strain obtained, and its wild-type compatible partner. Briefly, the mixture of the Δ*nrg1* strain in the *a2b2* background and the WT strain in the *a1b1* background was used to assess their virulence. This was compared with the infection when the mixture of the wild type stains FB1xFB2 was tested. The results obtained 14 days post-infection (dpi) are presented in Figure 8 as percentages of infected plants symptoms and their photographs. Observations of the infected plants indicate that when using the mixture of a *nrg1* mutant strain and the sexually compatible wild-type strain, a decrease in virulence was obtained compared to the plants inoculated with the wild-types mixture. There were more healthy plants surviving at the end of the study among the plants infected with the *nrg1* FB2 mutant, and their ability to form tumors was surprisingly reduced to almost 10%. These data indicate that the mutation is recessive, but with a quantitative effect, and accordingly, that deletion of the *NRG1* gene has substantial effects on virulence.

## 4. Discussion

pH is one of the environmental factors that most affects microbial cell growth and development. Environmental pH modifies membrane potential and affects micronutrient availability, protein function, and metal ion toxicity [50]. Fungi can tolerate acidic (pH 3) and alkaline (pH 9) extreme growth conditions, and depending on the external pH, they induce or repress genes involved in essential functions for their adaptation to the prevailing environmental conditions, ranging from metabolic adjustments to changes in morphology. In many cases, dimorphism in fungi is a crucial stage of their pathogenic cycle [51]. Although *U. maydis* is a well-studied model system, the roles played by the *NRG1* gene to overcome the effects of acid pH and other functions are still unknown.

Previously, we reported that the RIM101/PACC transcription factor regulates a large number of genes directly under alkaline conditions, but also that this regulation could be indirectly mediated through other transcription factors due to the pleiotropic response described in *U. maydis* [14,19]. This study shows how *NRG1* in *U. maydis* modulates a transcriptional response involving several processes, including virulence, and filamentous development.

From transcriptomic analyses, one of the represented functional categories was membrane transport; this was also reported previously in the RIM101 strain. Thus, a considerable number of genes related to membrane transport at alkaline pH were differentially expressed. In *C. albicans*, it is known that *NRG1* negatively regulates the expression of the calcium pump gene *PMR1* through binding to two motifs in its promoter; additionally, it plays a vital role in repressing specific genes (*HYR1*, *ALS8*, *HWPI* and *ECE1*) involved in hyphal development [4,6,7,52].

A further role of *NRG1* is related to the establishment of redox equilibrium into the fungal cell [53], affecting transcriptional regulation of *WCO1*. *WCO1* plays a role in at least two aspects: sensing light to control UV response [54] and possibly as a voltage sensor of altered ionic conditions generated by acid pH. It also may be working together with putative opsins homologs. There is no previous evidence of this kind of function for fungal models, but several reports of the use of two-component sensors in bacteria responding to acid pH have been reported. It was very interesting to find out that the possible Hog1 homolog expression gene was affected by the mutation of NRG1 in *U. maydis*.

Yeasts trigger an adaptive program in response to hyperosmotic stress that is mostly regulated by the high osmolarity glycerol (HOG) signaling pathway. The pathway core is the Hog1 MAP kinase (MAPK) cascade. Additionally, Hog1 is activated in response to a variety of stress stimuli, including oxidative stress, heavy metals and weak acids [55,56]. Thus, it can be deduced that this transcription factor could be regulating its expression to cover this cellular disturbance caused on the cell wall in *U. maydis*. This implies that a single MAP kinase coordinates various responses that are temporally, spatially and mechanistically different, agreeing with what has been reported in S. cerevisiae, where they have observed that the HOG pathway also responds to stresses such as oxidizing agents, heavy metals, weak acids and heat or cold shock [56]. Additionally, an interesting finding in our analysis is the overexpression of a probable homolog of Yap-like bZIP (UMAG_03296). Yap-like proteins regulate intracellular ROS levels by adjusting the antioxidant system activity; in *S. cerevisiae*, they detect defective levels of H_2_O_2_, and as a consequence, oxidant scavengers are induced to prevent cellular damage. Under these conditions, yeast cells activate the glutathione and thioredoxin pathways, which in turn activate Yap1 [57,58]. Moreover, Yap1 binds to promoter regions of genes coding for elements of the biosynthetic machinery [59], activating the osmotic regulator system by Hog1 [55]. Additionally, we found that the putative homolog of *Cry1* (UMAG_01131) and the probable homolog of the photolyase/cryptochrome family (UMAG_02144) both include a PHR domain involved in DNA-repair along with its sensory and regulatory activity [60,61].

We also found that, by means of a gene cluster for MEL biosynthesis expressed under nitrogen starvation conditions, *U. maydis* produces considerable amounts of extracellular glycolipids, such as ustilagic acid and other biosurfactants composed by mannosylerythritol lipids [39,47,62,63,64,65]. With exception of the Mat1 gene (UMAG_03114) that encodes an acetyltransferase, all the gene cluster for MEL biosynthesis is overexpressed in the mutant of *NRG1*; therefore, the FMA mutant could be an important biotechnological strain. We demonstrated that unlike FB2 and BMA2, FMA2 is able to produce MELs in the presence of nitrogen, which is a condition for the production of glycolipids not reported previously. It can be said, given MELs functions, that the transcription factor represses its production; since it is a very polar molecule, its absence could prevent negative flow to the outside and thus avoid loss of ions. Because biosurfactants are more soluble into the cell at acid pH, MEL enhances accessibility of hydrocarbon substrates by forming small emulsion droplets and increasing the surface area of the insoluble substrates [66]. Increased external osmolarity or ionic stress induces water efflux, high concentration of cytosolic ions (especially Na^+^) and cell shrinkage, which are harmful to cell growth [67]. Relating this phenotype to expression analysis, UMAG_02357 (putative mitogen-activated protein kinase HOG1) expression was affected by *nrg1* mutation. Some studies have related this gene function with cell wall adjustments related to osmotic stress adaptation and yeast-to-hyphae transition [68]. Moreover, from the promoter analysis, it was found that Rim101 and Nrg1 canonical binding sites are present on the 5’ DNA region of these open reading frames. In contrast to neutral pH conditions, cell growth at acid pH (pH 4) activates a battery of genes that seems to be related to cell Ca^+^, Na^+^, K^+^ and Mg^2+^ homeostasis. Among them, symporters, antiporters, transporters, carriers or permeases act as molecular devices that modulate substrate-specific equilibrium or translocation of solutes across the biological membrane. Major facilitator superfamily (MFS) is the largest family of secondary transporters, and targets a broad spectrum of substrates, including ions, carbohydrates, lipids, amino acids, peptides, nucleotides and small molecules [69]. We found that 15 MFS coding genes were differentially regulated by NRG1 under acid conditions, pointing out the importance of this cellular process in the cell. In the same way, it seems that repression of at least three different types of genes prevent the elevation of cellular oxidation (by repression of K, P-type ATPases) [14].

Another family of yeast activator protein (Yap) transcriptional factors found in all eukaryotes is involved in mediated stress responses, often associated with resistance to reactive oxygen species (ROS), osmotic imbalances, drugs or dangerous concentrations of heavy metals [70]. UMAG_11957 seems to encode a hypothetical histidine kinase (HK). It also contains several PAS domains in its structure and a phosphate acceptor domain, plus the response regulator receiver domain, bringing more evidence to the active role of two-component systems in this pH response for this particular Basidiomycota fungi. It might not be too daring to propose that acid pH, membrane voltage change or ionic strength stimuli induce the HK’s auto-phosphorylation. Phosphate is then transferred to a response regulator, which acts directly as a transcription factor to regulate a set of target genes or downstream elements such as mitogen-activated protein kinase (MAPK) cascades or cyclic adenosine monophosphate cAMP signaling systems required for the adaptation of acidic pH [71,72]. Most of these elements have been previously reported [12].

PHY1 gene (UMAG_05732) encodes a light-sensing protein. The output module contains an HK and the N-terminal response regulator (RR) domains [73]. This structure indicates that it could be acting as a two-component system that participates in the cellular processes of phosphorelay, which is related to membrane potential and virulence sensor. By now, it has only been reported that Phy1 participates in the fruiting bodies development in *U. maydis* [74]. We also confirmed the opsin1 and opsin2 function as outward rectifying proton pumps by highly elevated gene expression. Additionally, the putative codifying WCO1 is overexpressed. In *U. maydis*, it has been reported that White collar 1 protein encoded by UMAG_03180 acts as blue light photoreceptor involved in transcriptional regulation, contributes to UV-tolerance and basidiocarps development [54,74]. This protein contains two PAS domains. The nearest *N*-terminal PAS domain belongs to a subclass domain called LOV, a specialized type of light, oxygen, and voltage sensor. LOV domains are essential for photosensory protein light-sensing capabilities in plants, fungi and bacteria [75]. Along with this gene, the WCO2 act together to trigger the response to light. However, these homologs have roles beyond sensing light. In *Neurospora crassa*, the WC1/WC2 complex allows light to serve as an indicator of time-of-day, so it is part of the feedback loop during the rest of the day required for circadian timing [76]. In NRG1 *U. maydis* transcriptomic analyses, WCO1 was deregulated. Additionally, genes related to either elongation of the 1,3 ß-Glucan chains of cell wall proteins (UMAG_01640), *N*-acetylglucosamine (*NAG1*) synthesis pathway (UMAG_01716, UMAG_01718 and UMAG_04983) and chitin synthase 1 (UMAG_10718) are overexpressed. We believe that NAG elevation in the cell wall could function as a shell to protect against high anionic concentrations of acid conditions (see Supplementary Table S5). Our experimental results indicate that partial deletion of *NRG1* reduces virulence compared to the wild type mixture, reinforced by the finding that *NRG1* represses virulence-involved genes at acid conditions. These repressed genes possibly include proteins for multidrug resistance, siderophore biosynthesis such as the Mig 2–5 codifying gene, and Pit2*, a cysteine protease inhibitor whose target are part of the plant defense response (see Supplementary Table S5).

**Figure 9:**
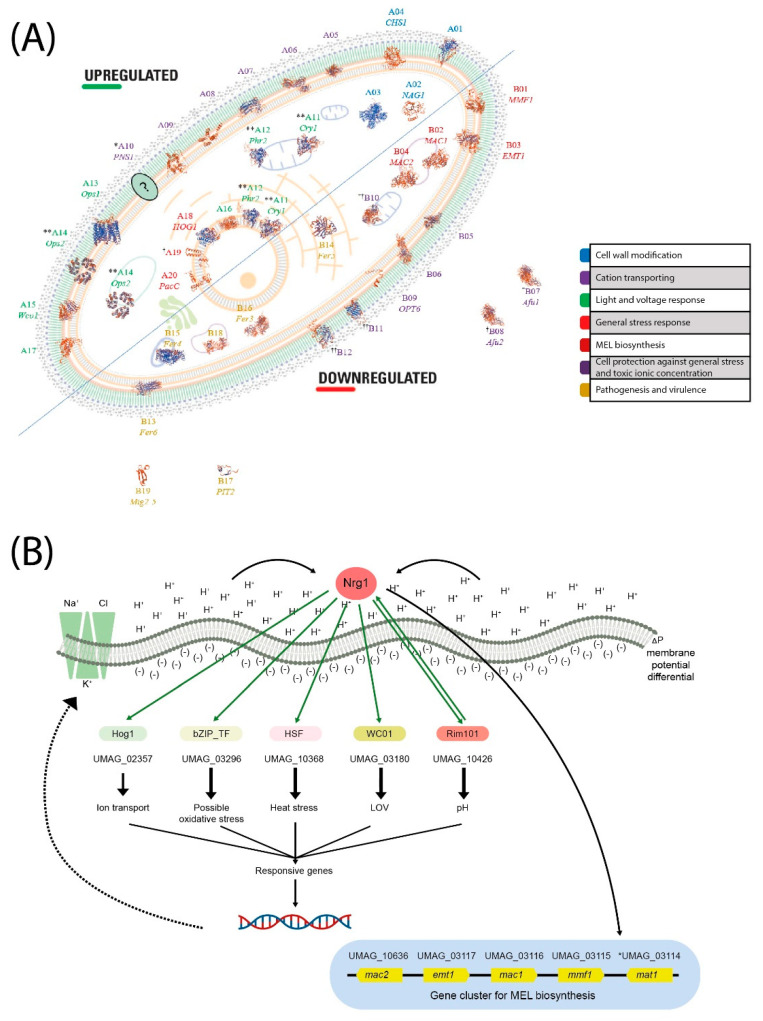
Analytical interpretation of the enrichment of transcriptomic analysis of *NRG1* at acid pH in the Basidiomycota *U. maydis*. (**A**) Subcellular localization of regulated proteins by *Nrg1*. The localization of proteins is shown based on gene ontology, sequence similarity and curator. The protein structure was modeled in ExPASy tool swiss model platform available in www.swissmodel.expasy.org/interative. (**B**) Nrg1 function; in an acidic pH environment, the transcription factor *Nrg1* regulates the expression of another pH regulator *RIM101*, the photoreceptor *WCO1*, a bZIP transcription factor, a putative mitogen-activated protein kinase *HOG1*, a gene cluster for MEL biosynthesis and others. Growth in the acidic pH environment of *U. maydis*, cells activate a concerted response, while the external cation-rich zone (pH 4) forms a membrane potential difference; these conditions induce the exchange of K^+^, Na^+^ and Cl^−^ ions and a transcriptomic response.

Comprehensive in silico promoter analysis of the regulated genes and the transcriptome of the differential genes (Supplementary Table S6) allowed us to structure a tentative functional model. Most relevant results can be seen in Figure 9. Based on this model, we point out the most relevant UmNRG1 roles in the acid pH response, including several cellular functions and structures.

## 5. Conclusions

In summary, we have identified the transcription factor Nrg1 in the Basidiomycota fungus *U. maydis* and proceeded to mutate its gene to determine its functional roles. Our results indicate that the Nrg1 protein plays a significant role in several cellular processes, including the response to acid pH, morphogenesis and virulence.

## Figures and Tables

**Table 1:** Strains of *Ustilago maydis* used in this study.

Organism	Strain	Genotype	Source
*U. maydis* (WT)	FB1	*a1b1*	Banuett and Herskowitz [20]
*U. maydis* (WT)	FB2	*a2b2*	Banuett and Herskowitz [20]
*U. maydis*	BMA2	*a2b2*, ∆*rim101*	Aréchiga-Carvajal and Ruiz-Herrera [19]
*U. maydis*	FMA2	*a2b2*, ∆*nrg1*	This work

## Supplementary Materials

The following are available online at https://www.mdpi.com/2309-608X/7/2/91/s1, Figure S1: Wild type and mutant strain cell size, Table S1: List of primers used for *NRG1* mutation in *U. maydis*, Table S2: List of oligonucleotides used for RT_qPCR, Table S3: DEGs at pH4 for *NRG1* strain, Table S4: Transcription analysis RT_qPCR, Table S5: Upregulated and down regulated genes by *NRG1*, Table S6: NRG motifs.

## References

[1] Cervantes-Chávez J.A., Ortiz-Castellanos L., Tejeda-Sartorius M., Gold S., Ruiz-Herrera J. (2010). Functional analysis of the pH responsive pathway Pal/Rim in the basidiomycete *Ustilago maydis*. Fungal Genet. Biol..

[2] Sartorel E., Perez-Martin J. (2012). The distinct interaction between cell cycle regulation and the widely conserved morphogenesis-related (MOR) pathways in the fungus *Ustilago maydis* determines morphology. J. Cell Sci..

[3] Cervantes-Montelongo J.A., Aréchiga-Carvajal E.T., Ruiz-Herrera J. (2017). Adaptation of *Ustilago maydis* to extreme values: A transcriptomic analysis. J. Basic Microbiol..

[4] Vyas A., Berkey C.D., Miyao T., Carlson M. (2005). Repressors Nrg1 and Nrg2 regulate a set of stress-responsive genes in *Sacharomyces cerevisiae*. Eukaryot. Cell.

[5] Park S.H., Koh S.S., Chun J.H., Hwang H.J., Kang H.S. (1999). Nrg1 is a transcriptional repressor for glucose repression of STA1 gene expression in *Saccharomyces cerevisiae*. Mol. Cell. Biol..

[6] Braun B.R., Kadosh D., Johnson A.D. (2001). NRG1, a repressor of filamentous growth in *C. albicans*, is down-regulated during filament induction. EMBO J..

[7] Murad A.M., d’Enfert C., Gaillardin C., Tournu H., Tekaia F., Talibi D., Marechal D., Marchais V., Cottin J., Brown A.J. (2001). Transcript profiling in *Candida albicans* reveals new cellular functions for the transcriptional repressors CaTup1, CaMig1 and CaNrg1. Mol. Microbiol..

[8] Rocha C.R., Schroppel K., Harcus D., Marcil A., Dignard D., Taylor B.N., Thomas D.Y., Whiteway M., Leberer E. (2001). Signaling through adenylyl cyclase is essential for hyphal growth and virulence in the pathogenic fungus *Candida albicans*. Mol. Biol. Cell.

[9] Bockmühl D.P., Krishnamurthy S., Gerads M., Sonneborn A., Ernst J.F. (2001). Distinct and redundant roles of the two protein kinase A isoforms Tpk1p and Tpk2p in morphogenesis and growth of *Candida albicans*. Mol. Microbiol..

[10] Lu Y., Su C., Wang A., Liu H. (2011). Hyphal development in *Candida albicans* requires two temporally linked changes in promoter chromatin for formation and maintenance. PLoS Biol..

[11] Lu Y., Su C., Liu H. (2014). *Candida albicans* hyphal initiation and elongation. Trends Microbiol..

[12] Cramer K.L., Gerrald Q.D., Nichols C.B., Price M.S., Alspaugh J.A. (2006). Transcription factor Nrg1 mediates capsule formation, stress response, and pathogenesis in *Cryptococcus neoformans*. Eukaryot. Cell.

[13] Kämper J., Kahmann R., Bölker M., Ma L., Brefort T., Saville B.J., Banuett F., Kronstad J.W., Gold S.E., Müller O. (2006). Insights from the genome of the biotrophic fungal plant pathogen *Ustilago maydis*. Nature.

[14] Franco-Frías E., Ruiz-Herrera J., Aréchiga-Carvajal E.T. (2014). Transcriptomic analysis of the role of Rim101/PacC in the adaptation of *Ustilago maydis* to an alkaline environment. Microbiology.

[15] Ruiz-Herrera J., León-Ramírez C.G., Guevara-Olvera L., Cárabez-Trejo A. (1995). Yeast-mycelial dimorphism of haploid and diploid strains of *Ustilago maydis*. Microbiology.

[16] Davis D.A. (2009). How human pathogenic fungi sense and adapt to pH: The link to virulence. Curr. Opin. Microbiol..

[17] Martínez-Espinoza A.D., Ruiz-Herrera J., León-Ramírez C.G., Gold S.E. (2004). MAP kinase and cAMP signaling pathways modulate the pH-induced yeast-to-mycelium dimorphic transition in the corn smut fungus *Ustilago maydis*. Curr. Microbiol..

[18] Peñalva M.A., Arst H.N. (2004). Recent advances in the characterization of ambient pH regulation of gene expression in filamentous fungi and yeast. Annu. Rev. Microbiol..

[19] Aréchiga-Carvajal E.T., Ruiz-Herrera J. (2005). The RIM101/PacC homologue from the basidiomycete *Ustilago maydis* is functional in multiple pH-sensitive phenomena. Eukaryot. Cell.

[20] Banuett F., Herskowitz J. (1989). Different *a* alleles of *Ustilago maydis* are necessary for maintenance of filamentous growth but not for meiosis. Proc. Natl. Acad. Sci. USA.

[21] Holliday R., King R.C. (1974). Ustilago maydis. Handbook of Genetics.

[22] García-Pedrajas M.D., Nadal M., Kapa L.B., Perlin M.H., Andrews D.L., Gold S.E. (2008). DelsGate, a robust and rapid gene deletion construction method. Fungal Genet. Biol..

[23] Chavez-Ontiveros J., Martinez-Espinoza A., Ruiz-Herrera J. (2000). Double chitin synthetase mutants from the corn smut fungus *Ustilago maydis*. New Phytol..

[24] Hoffman C.S., Wriston F. (1987). A ten-minute DNA preparation from yeast efficiently releases autonomous plasmids for transformation of *Escherichia coli*. Gene.

[25] Yu J.H., Hamari Z., Han K.H., Seo J.A., Reyes-Dominguez Y., Scazzocchio C. (2004). Double-joint PCR: A PCR-based molecular tool for gene manipulations in filamentous fungi. Fungal Genet. Biol..

[26] Tsukuda T., Carleton S., Fotheringham S., Holloman W.K. (1988). Isolation and characterization of an autonomously replicating sequence from *Ustilago maydis*. Mol. Cell. Biol..

[27] Sambrook J., Russell J.W. (2001). Molecular Cloning: A Laboratory Manual.

[28] Edgar R., Domrachev M., Lash A.E. (2002). Gene expression omnibus: NCBI gene expression and hybridization array data repository. Nucleis Acids Res..

[29] Huber W., Carey V.J., Gentleman R., Anders S., Carlson M., Carvalho B.S., Bravo H.C., Davis S., Gatto L., Girke T. (2015). Orchestrating high-throughput genomic analysis with Bioconductor. Nat. Methods.

[30] Götz S., Garcia-Gómez J.M., Terol J., Willians T.D., Nagaraj S.H., Nueda M.J., Robles M., Talón M., Dopazo J., Conesa A. (2008). High-throughput functional annotation and data mining with the Blast2GO suite. Nucleic Acids Res..

[31] Gaidatzis D., Lerch A., Hahne F., Stadler M.B. (2015). QuasR: Quantification and annotation of short reads in R. Bioinformatics.

[32] Langmead B., Trapnell C., Pop M., Salzberg S.L. (2009). Ultrafast and memory-efficient alignment of short DNA sequences to the human genome. Genome Biol..

[33] Au K.F., Jiang H., Lin L., Xing Y., Wong W.H. (2010). Detection of splice junctions from paired-end RNA-seq data by SpliceMap. Nucleic Acids Res..

[34] Szklarczyk D., Gable A.L., Lyon D., Junge A., Wyder S., Huerta-Cepas J., Simonovic M., Doncheva N.T., Morris J.H., Bork P. (2019). STRING v11: Protein–protein association networks with increased coverage, supporting functional discovery in genome-wide experimental datasets. Nucleic Acids Res..

[35] León-Ramírez C.G., Cabrera-Ponce J.L., Martínez-Soto D., Sánchez-Arreguin J.A., Aréchiga-Carvajal E.T., Ruiz-Herrera J. (2017). Transcriptomic analysis of basidiocarp development in *Ustilago maydis* (DC) Cda. Fungal Genet. Biol..

[36] R Core Team (2013). R: A Language and Environment for Statistical Computing.

[37] Monteiro P.T., Mendes N.D., Teixeira M.C., d’Orey S., Tenreiro S., Mira N.P., Pais H., Francisco A.P., Carvalho A.M., Lourenço A.B. (2008). YEASTRAC-DISCOVERER: New tools improve the analysis of transcriptional regulatory associations in *Saccharomyces cerevisiae*. Nucleic Acids Res..

[38] Teixera M.C., Monteiro P.T., Palma M., Costa C., Godinho C.P., Pais P., Cavalheiro M., Antunes M., Lemos A., Pedreira T. (2018). YEASTRACT: An upgraded database for the analysis of transcription regulatory networks in *Saccharomyces cerevisiae*. Nucleic Acids Res..

[39] Hewald S., Josephs K., Bölker M. (2005). Genetic analysis of biosurfactant production in *Ustilago maydis*. Appl. Environ. Microbiol..

[40] Nern A., Arkowitz R.A. (2002). A GTP-exchange factor required for cell orientation. Nature.

[41] Wedlich-Soldner R., Wai S.C., Schmidt T., Li R. (2004). Robust cell polarity is a dynamic state established by coupling transport and GTPase signaling. J. Cell Biol..

[42] Castresana J., Sarastre M. (1995). Does Vav bind to F-actin through a CH domain?. FEBS Lett..

[43] Vancompernolle K., Gimona M., Herzog M., Damme J., Vandekerckhove J., Small V. (1990). Isolation and sequence of a tropomyosin-binding fragment of turkey gizzard calponin. FEBS Lett..

[44] Kolakowski J., Makuch R., Stepkowski D., Dabrowska R. (1995). Interaction of calponin with actin its functional implications. Biochem. J..

[45] Bassiliana M., Hopkins J., Arkowitz R.A. (2005). Regulation of the Cdc42/Cdc24 GTPase module during *Candida albicans* hyphal growth. Eukaryot. Cell.

[46] Brombacher K., Fisher B.B., Rüfenacht K., Eggen R.I. (2006). The role of Yap1p and Skn7p-mediated oxidative stress response in the defense of *Saccharomyces cerevisiae* against singlet oxygen. Yeast.

[47] Hewald S., Linne U., Sherer M., Marahiel M.A., Kämper J., Bölker M. (2006). Identification of a gene cluster for Biosynthesis of mannosylerythritol lipids in the Basidiomycetous fungus *Ustilago maydis*. Appl. Environ. Microbiol..

[48] Ruepp A., Zollner A., Maier D., Alberman K., Hani J., Mokrejs M., Tetko I., Güldener U., Mannhaupt G., Münsterkö M. (2004). The Funcat, a functional annotation scheme for systematic classification of proteins from whole genomes. Nucleic Acids Res..

[49] Livak K.J., Schmittgen T.D. (2001). Analysis of relative gene expression data using real-time quantitative PCR and the 2∆∆CT method. Methods.

[50] Selvig K., Aspaugh J.A. (2011). pH response pathways in fungi: Adapting to host-derived and environmental signals. Microbiology.

[51] Su C., Yu J., Lu Y. (2018). Hyphal development in *Candida albicans* from different cell states. Curr. Genet..

[52] Zhao Y., Du J., Xiong B., Xu H., Jiang L. (2013). ESCRT components regulate the expression of the ER/Golgi calcium pump gene *PMR1* through the Rim101/Nrg1 pathway in budding yeast. J. Mol. Cell Biol..

[53] Cotter P.D., Hill C. (2003). Surviving the acid test: Responses of gram-positive bacteria to low pH. Microbiol. Mol. Biol. Rev..

[54] Brych A., Mascarenhas J., Jaeger E., Charkiewicz E., Pokorny R., Bölker M., Doehlemann G., Batschauer A. (2016). White collar 1-induced photolyase expression contributes to UV-tolerance of *Ustilago maydis*. Microbiologyopen.

[55] Bilsland E., Molin C., Swaminathan S., Ramne A., Sunnerhagen P. (2004). Rck1 and Rck2 MAPKAP kinases and the HOG pathway are required for oxidative stress resistance. Mol. Microbiol..

[56] Smith D.A., Morgan B.A., Quinn J. (2010). Stress signaling to fungal stress-activated protein kinase pathways. FEMS Microbiol. Lett..

[57] Reverberi M., Gazzetti K., Punelli F., Scarpari M., Zjalic S., Ricelli A., Fabbri A.A., Fanelli C. (2012). Aoyap1 regulates OTA synthesis by controlling cell redox balance in *Aspergillus ochraceus*. Appl. Microbiol. Biotechnol..

[58] Toledano M.B., Delaunay A., Monceau L., Tacnet F. (2004). Microbial H_2_O_2_ sensors as archetypical redox signaling modules. Trends Biochem. Sci..

[59] Yin W.B., Amaike S., Wohlbach D.J., Gash A.P., Chiang Y.M., Wang C.C., Bok J.W., Rohlfs M., Keller N.P. (2012). An *Aspergillus nidulans* bZIP response pathway hardwired for defensive secondary metabolism operates through *aflR*. Mol. Microbiol..

[60] Bayram O., Biesemann C., Krappmann S., Galland O., Braus G.H. (2008). More than a repair enzyme: *Aspergillus nidulans* photolyase-like CryA is a regulator of sexual development. Mol. Biol. Cell.

[61] García-Esquivel M., Esquivel-Naranjo E.U., Hernández-Oñate M.A., Ibarra-Laclette E., Herrera-Estrella A. (2016). The *Trichoderma atroviride* cryptochrome/photolyase genes regulate the expression of blr1-independent genes both in red and blue light. Fungal Biol..

[62] Kurz M., Eder C., Isert D., Li Z., Paulus E.F., Schiell M., Toti L., Vértesy L., Wink J., Seibert G. (2003). Ustilipids, acylated β-d-mannopynanosyl d-erythritols from *Ustilago maydis* and *Geotrichum candidum*. J. Antibiot..

[63] Lemieux R.U. (1953). Biochemistry of the ustilaginales: VIII. the structures and configurations of the ustilic acids. Can. J. Chem..

[64] Fluharty A.L., O’Brien J.S. (1969). A mannose- and erythritol-containing glycolipid from *Ustilago maydis*. Biochemistry.

[65] Spoeckner S., Wray V., Nimtz M., Lang S. (1999). Glycolipids of the smut fungus *Ustilago maydis* from cultivation on renewable resources. Appl. Microbiol. Biotechnol..

[66] Ron E.Z., Rosenberg E. (2001). Natural roles of biosurfactants. Environ. Microbiol..

[67] Wood J.M. (2011). Bacterial osmoregulation: A paradigm for the study of cellular homeostasis. Annu. Rev. Microbiol..

[68] Rzechonek D.A., Day A.M., Quinn J., Mironczuk A.M. (2018). Influence of ylHog1 MAPK kinase on *Yarrowia lipolytica* stress response and erythritol production. Sci. Rep..

[69] Reddy V.S., Shlykov M.A., Castillo R., Sun E.I., Saier M.H. (2012). The major facilitator superfamily (MFS) revisited. FEBS J..

[70] Yin W.B., Reinke A.W., Szilágil M., Emri T., Chiang Y.M., Keating A.E., Pócsi I., Wang C.C., Keller N.P. (2013). bZIP transcription factors affecting secondary metabolism, sexual development and stress responses in *Aspergillus nidulans*. Microbiology.

[71] Hérivaux A., So Y.S., Gastebois A., Latgé J.P., Bouchara J.P., Bahn Y.S., Papon N. (2016). Major sensing proteins in pathogenic fungi: The hybrid histidine kinase family. PLoS Pathog..

[72] Kabbara S., Hérivaux A., Dugé de Bernonville T., Courdavault V., Clastre M., Gastebois A., Osman M., Hamze M., Cock J.M., Schaap P. (2018). Diversity and evolution of sensor histidine kinases in eukaryotes. Genome Biol. Evol..

[73] Purschwitz J., Müller S., Fischer R. (2009). Mapping the interaction sites of *Aspergillus nidulans* phytochrome FphA with the global regulator VeA and the White collar proteins LreB. Mol. Genet. Genom..

[74] Sánchez-Arreguin J.A., Cabrera-Ponce J.L., León-Ramírez C.G., Camargo-Escalante M.O., Ruiz-Herrera J. (2020). Analysis of the photoreceptors involved on light-depending basidiocarp formation in *Ustilago maydis*. Arch. Microbiol..

[75] Briggs W.R., Spudich J.L. (2005). Handbook of Photosensory Receptors.

[76] Baker C.L., Loros L.L., Dunlap J.C. (2012). The circadian clock of *Neurospora crassa*. FEMS Microbiol. Rev..

