# Supplementary material for: Acid pH Strategy Adaptation through NRG1 in Ustilago maydis
Source: J Fungi (Basel). 2021 Jan 28;7(2):91. doi: 10.3390/jof7020091 (PMC7912220; doi:10.3390/jof7020091)
Supplement: Supplementary file 1 [file jof-07-00091-s001.zip › Supplementary files/Table S1 List of primers used for NRG1 mutation in U. maydis.docx]

**Supplementary Table 1.** List of primers used in this study for NRG1 mutation in *U. maydis*. Chimerical primers contain a complementary region to 5´ and 3´ ends of the carboxin resistance gene used as selection marker and were designed to fuse gene and cassette sequences in vitro (NRG1-BCbx and NRG1-CCbx).

| Primer | Orientation | Tm(ºC) | Sequence (5´-3´) |
| --- | --- | --- | --- |
| NRG1-A | Forward | 69.1 | cgt cat tct ctc gcc aat cct c |
| NRG1-BCbx | Reverse | 88.8 | ccc cac ctt ctt ccg cga tct tcc gaa ttt gtt tgc tcg gtc ttg tac g |
| NRG1-CCbx | Forward | 87.9 | gta cga aag cga gac gag ttg agc gac gtg tgt gcg gac agt gaa gag tat gtc gg |
| NRG1-D | Reverse | 69.6 | caa tac gga gga tga ggt gac gag |
| NRG1-E | Forward | 64.3 | ggt tat cac ctt gta cac ccc tc |
| NRG1-F | Reverse | 62.9 | cat tcg cac cat gta gat cc |
| NRG1-RT1 | Forward | 66.0 | ctt gac cac ctc gac ctt gac |
| NRG1-Rsouth | Reverse | 70.1 | gat ttg ggg ctc tgc gat gag |
| Cbx-1 | Forward | 62.3 | cgg aag atc gcg gaa gaa g |
| Cbx-2 | Reverse | 62.3 | tcg ctc aac tcg tct cgc t |
