# Supplementary material for: Acid pH Strategy Adaptation through NRG1 in Ustilago maydis
Source: J Fungi (Basel). 2021 Jan 28;7(2):91. doi: 10.3390/jof7020091 (PMC7912220; doi:10.3390/jof7020091)
Supplement: Supplementary file 1 [file jof-07-00091-s001.zip › Supplementary files/Table S2 List of oligonucleotides used for RT_qPCR.docx]

**Supplementary Table 2.** Genes and primers used for quantitative RT-PCR analysis, of selected differential expressed genes at pH4 of *∆nrg1* mutant.

| Gene | Primer name | Sequence |
| --- | --- | --- |
| UMAG_06217 (Actin)* | ActinF | 5´-CTTGCCCCTAGCTCGATGAAGG- 3´ |
|  | ActinR | 5´ -CTCCTGCTTCGAGATCCACATC- 3´ |
| UMAG_02357 | 2357for | 5´-CATCCCTACCTCGCTCCTTAC-3´ |
|  | 2357rev | 5´-CTCGTCTTGACCCGAATTGTC-3 |
| UMAG_06085 | UM06085F | 5´-ACGTCTGTTGCTTGTTGTGC-3´ |
|  | UM06085R | 5´-AGTGACCCAGAGCAGGAAG-3´ |
| UMAG_00056 | UM00056F | 5´-GCTGGAACACGATCCTTAGC-3´ |
|  | UM00056R | 5´-GAAGCGTAAGTTCCGCAGAC-3 |
| UMAG_03382 | UM03382F | 5´-TTACACACGACACCCAGCTC-3´ |
|  | UM03382R | 5´-GCCTGTCCCTTCTCGGATAG-3´ |
| UMAG_10242 | UM10242F | 5´-AGTCCACCCAAGTGATGTCC-3´ |
|  | UM10242R | 5´-GCTTTGTATGGCTCGGTCTC-3´ |
| UMAG_11506 | UM11506F | 5´-GGAAACAGACGAGCATGGAT-3´ |
|  | UM11506R | 5´-GTCATCAGCACCTGTCCTTG-3´ |
| UMAG_15036 | Nrg1F | 5´-ACTTGACCACCTCGACCTTG-3´ |
|  | Nrg1R | 5´-AAAGGCGACACCATTCAATC-3´ |
| UMAG_10426 | RimFb2F | 5´-GACTCGCACATGGCTGAGAGG-3´ |
|  | RimFb2R | 5´-GCCGAGGGATCAAAGGCGG-3´ |
| UMAG_03180 | 3180for | 5´-GACAGTTCCTCATATCCAGATG-3´ |
|  | 3180rev | 5´-GAAATCAGTACTGTGACCAGC-3´ |

*Gene used as internal control.
