# Supplementary material for: Acid pH Strategy Adaptation through NRG1 in Ustilago maydis
Source: J Fungi (Basel). 2021 Jan 28;7(2):91. doi: 10.3390/jof7020091 (PMC7912220; doi:10.3390/jof7020091)
Supplement: Supplementary file 1 [file jof-07-00091-s001.zip › Supplementary files/Table S4 Transcription analysis RT_qPCR.docx]

**Supplementary Table 4.** Transcription analysis of selected genes by quantitative RT-PCR.

| ID Gene | Gene description | Fold change RNA_Seq analysis | Fold change quantitative real-time PCR analysis |
| --- | --- | --- | --- |
| UMAG_00056 | Amino acid permease | 5.94 | 2.71 |
| UMAG_02357 | Mitogen-activated protein kinase HOG1 | -2.32 | -3.21 |
| UMAG_03180 | White collar 1a | -2.53 | -3.10 |
| UMAG_03382 | Related to 3-phytase A precursor | 3.97 | 5.06 |
| UMAG_06085 | Hypothetical protein | 2.49 | 3.07 |
| UMAG_10242 | Hypothetical protein | 2.44 | 8.6 |
| UMAG_10426 | Transcription factor pacC | 3.12 | 4.56 |
| UMAG_11506 | Hypothetical protein | 20.18 | 11.01 |

Expression values obtained by qRT-PCR for selected genes (UMAG_00056, UMAG_02357, UMAG_03180, UMAG_03382, UMAG_06085, UMAG_10242, UMAG_10426 and UMAG_11506), samples obtained from cells grown in minimum medium at pH 4 after 24 h at 28°C. Expression levels shown in Log scale as RQ (relative quantification number) or 2^^-ΔΔCT^. WT strain was used as calibrator sample, therefore, ΔΔCT was obtained from the equation ΔCT(WT)–ΔCT (of each evaluated sample), where ΔCT represent[CT(selected genes)*E]-[CT(Actin)*E], E is the PCR efficiency estimated as ([10^(-1/m)]-1).
