# Supplementary material for: Acid pH Strategy Adaptation through NRG1 in Ustilago maydis
Source: J Fungi (Basel). 2021 Jan 28;7(2):91. doi: 10.3390/jof7020091 (PMC7912220; doi:10.3390/jof7020091)
Supplement: Supplementary file 1 [file jof-07-00091-s001.zip › Supplementary files/Table S5 Upregulated and down regulated genes by NRG1.docx]

**Supplementary Table 5a.** Upregulated gene enrichment by NRG1 at acid pH.

| **Cell strategy** | **Gene ID** | **Code** | **Function** | **Global description** | **Reference** |
| --- | --- | --- | --- | --- | --- |
| Cell wall modification | UMAG_01640 | A01 | 1,3-beta-glucanosyltransferase. 1,3-beta-glucan chains elongation | Probably cell wall thickness increases by elevation of NAG1 as a response to mantain stability and counter high H^+^ ions outside the cell wall | Ma *et al*., 2008; Gunasekera *et al*., 2010; Sintu & Sudip, 2012 |
|  | UMAG_01716 | A02 | Hypothetical protein. N-acetylglucosamine-induced protein which increases resistance to chitin synthase inhibitors |  |  |
|  | UMAG_01718 | A03 | Glucosamine-6-phosphate isomerase related in N-acetylglucosamine pathway. The elevation of NAG1 can be related to the augmentation of N-acetylglucosamine |  |  |
|  | UMAG_10718 | A04 | Chitin synthase 1. Plays a major role in cell wall biogenesis |  |  |
| Cation transporting | UMAG_00204 | A05 | Na^+^ / K^+^ P-type ATPase | Apparently there is a genetic regulation by methyltransferases of specific proteins including calmodulin and Hsp70, adjusting the concentration of cations outside the cell wall through solute transport in response to ion gradient | Rappas *et al*., 2004; Parks *et al*., 2019 |
|  | UMAG_15043 | A06 | Putative Na^+^ / K^+^ P-type ATPase |  |  |
|  | UMAG_03470 | A07 | Ca^2+^-transporting ATPase. Magnesium dependent enzyme |  |  |
|  | UMAG_04923 | A08 | Ca^2+^ antiporter |  |  |
|  | UMAG_02504 | A09 | Putative ionic channel regulatory protein of the major superfamily of facilitators |  |  |
| Light and voltage response | UMAG_01131 | A10 | Photolyase/cryptochrome family Cry1. Protects and repairs DNA | Apparently cell is sensing voltage exchange derived as a consequence of acid pH outside cell wall by signal transduction and specific gene response expression | Brych *et* *al*., 2016; Panzer *et* *al*., 2019; Sánchez-Arreguin *et* *al*., 2019 |
|  | UMAG_02144 | A11 | Photolyase/cryptochrome family Phr2. Protects and repairs DNA |  |  |
|  | UMAG_02629 | A12 | Vacuolar photoactive opsin Ops1 |  |  |
|  | UMAG_00371 | A13 | Vacuolar photoactive opsin Ops2 |  |  |
|  | UMAG_11957 | A14 | Sensor and signal transduction PAS containing domain |  |  |
|  | UMAG_03180 | A15 | Signal sensor domain Wco1 |  |  |
|  | UMAG_06287 | A16 | Lycopene cyclase phytoene synthase |  |  |
| General stress response | UMAG_02357 | A17 | Mitogen-activated protein kinase HOG1 | It seems that under acid pH, transcription factors activate and regulate the expression of specific genes | Cervantes-Montelongo *et* *al*., 2016; Rzechonek *et* *al*., 2018 |
|  | UMAG_03296 | A18 | Putative transcription fator with basic-leucine zipper domain (bZIP) |  |  |
|  | UMAG_10426 | A19 | pH-response transcription factor pacC/Rim101 |  |  |

**Supplementary Table 5b.** Downregulated gene enrichment by NRG1 at acid pH.

| **Cell strategy** | **Gene ID** | **Code** | **Function** | **Global description** | **Reference** |
| --- | --- | --- | --- | --- | --- |
| MEL biosynthesis | †UMAG_03115 | B01 | Major Facilitator invovled in MEL transport in response to chemiosmotic ion gradient | This cluster are repressed by NRG1 due MEL increases heavy metals bioavailability and can disrupt cytoplasmic membranes leading to cell lysis | Hewald *et al*., 2005, 2006; Arutchelvi *et* *al*., 2008; Saika *et* *al*., 2016 |
|  | †UMAG_03116 | B02 | Acyltransferase invovled in MEL production, also involved in mycotoxin biosynthesis |  |  |
|  | †UMAG_03117 | B03 | Erythritol-mannosyl-transferase invovled in MEL production |  |  |
|  | †UMAG_10636 | B04 | Acyltransferase invovled in MEL production |  |  |
| Cell protection against general stress and toxic ionic concentration protection | UMAG_06433 | B05 | K, P-Type ATPase, Cation ATPase | Cation ATPase represión apparently prevent intracelular oxidation, also, it seems like O-glycosyl bonds are weak in acid pH and are exchanged by N-glycosylation | Gilead & Shoham, 1995; Lubkowitz *et* *al*., 1997; de la Cruz *et* *al*., 2003; Fujitani *et* *al*., 2003; Segall *et* *al*., 2003 |
|  | UMAG_05038 | B06 | K, P-Type ATPase, Cation ATPase |  |  |
|  | UMAG_01829 | B07 | Arabinofuranosidase 1, catalyzes the hydrolysis of alpha-L-arabinofuranoside |  |  |
|  | UMAG_00837 | B08 | Arabinofuranosidase 2, involved in amino sugar and nucleotide metabolism, hydrolyzes O-glicosyl and S-glicosyl compounds |  |  |
|  | UMAG_04347 | B09 | Oligopeptide transporter 6, oligopeptide transmembranal transporter |  |  |
|  | UMAG_04106 | B10 | O-methyltransferase. Have domain which might be universal among S-adenosyl-L-methionine (AdoMet)-dependent methyltransferases |  |  |
|  | UMAG_01898 | B11 | Hypothetical protein with hydrolase activity wich catalyzes O-glycosyl bonds, involved in carbohydrates metabolism |  |  |
|  | UMAG_04368 | B12 | Hypothetical protein with hydrolase activity wich catalyzes O-glycosyl bonds hydrolysis, involved in carbohydrates metabolism |  |  |
| Pathogeneis and virulence | *UMAG_01431 | B13 | Multidrug resistance protein of gene cluster involved in siderophore biosynthesis for virulence | Apparently repression of this genes, reduces virulence and pathogenesis mediated by: represión of energy supplier for appressorium formation, siderophore formation and inhibition of plant defense by inhibitor of cystein proteases, acting directly in tumor formation and decressing chlorosis and anthrachnosis as a result of Mig 2-5 protein repression | Basse *et* *al*., 2002; Farfsing *et* *al*., 2005; Winterberg *et* *al*., 2010; Doehlemann *et* *al*., 2011; Mueller *et* *al*., 2013; Lanver *et* *al*., 2014 |
|  | *UMAG_01432 | B14 | Multidrug resistance protein of gene cluster involved in siderophore biosynthesis for virulence |  |  |
|  | *UMAG_01433 | B15 | Multidrug resistance protein of gene cluster involved in siderophore biosynthesis for virulence |  |  |
|  | *UMAG_01434 | B16 | Multidrug resistance protein of gene cluster involved in siderophore biosynthesis for virulence |  |  |
|  | *UMAG_01375 | B17 | Cysteine-protease inhibitor, PIT2 inhibits cysteine proteases which activity is related with plant defense respons and tumor formation |  |  |
|  | *UMAG_05422 | B18 | Fatty acyl-CoA reductase, catalyzes fatty acyl-CoA reduction |  |  |
|  | *UMAG_06181 | B19 | Mig2-5 protein, involved in pathogenesis and virulence |  |  |

**†** MEL biosynthesis gene cluster

***** Siderophore gene cluster

1. Arutchelvi, J. I., Bhaduri, S., Uppara, P. V., & Doble, M. (2008). Mannosylerythritol lipids: a review. *Journal of industrial microbiology & biotechnology*, *35*(12), 1559-1570.
2. Basse, C. W., Kolb, S., & Kahmann, R. (2002). A maize‐specifically expressed gene cluster in Ustilago maydis. *Molecular microbiology*, *43*(1), 75-93.
3. Brych, A., Mascarenhas, J., Jaeger, E., Charkiewicz, E., Pokorny, R., Bölker, M., ... & Batschauer, A. (2016). White collar 1‐induced photolyase expression contributes to UV‐tolerance of Ustilago maydis. *Microbiologyopen*, *5*(2), 224-243.
4. Cervantes‐Montelongo, J. A., Aréchiga‐Carvajal, E. T., & Ruiz‐Herrera, J. (2016). Adaptation of Ustilago maydis to extreme pH values: A transcriptomic analysis. *Journal of basic microbiology*, *56*(11), 1222-1233.
5. de la Cruz, I. P., Levin, J. Z., Cummins, C., Anderson, P., & Horvitz, H. R. (2003). sup-9, sup-10, and unc-93 may encode components of a two-pore K+ channel that coordinates muscle contraction in Caenorhabditis elegans. *Journal of Neuroscience*, *23*(27), 9133-9145.
6. Doehlemann, G., Reissmann, S., Aßmann, D., Fleckenstein, M., & Kahmann, R. (2011). Two linked genes encoding a secreted effector and a membrane protein are essential for Ustilago maydis‐induced tumour formation. *Molecular microbiology*, *81*(3), 751-766.
7. Farfsing, J. W., Auffarth, K., & Basse, C. W. (2005). Identification of cis-active elements in Ustilago maydis mig2 promoters conferring high-level activity during pathogenic growth in maize. *Molecular plant-microbe interactions*, *18*(1), 75-87.
8. Fujitani, N., Kanagawa, M., Aizawa, T., Ohkubo, T., Kaya, S., Demura, M., & Nitta, K. (2003). Structure determination and conformational change induced by tyrosine phosphorylation of the N-terminal domain of the α-chain of pig gastric H+/K+-ATPase. *Biochemical and biophysical research communications*, *300*(1), 223-229.
9. Gilead, S., & Shoham, Y. (1995). Purification and characterization of alpha-L-arabinofuranosidase from Bacillus stearothermophilus T-6. *Appl. Environ. Microbiol.*, *61*(1), 170-174.
10. Gunasekera, A., Alvarez, F. J., Douglas, L. M., Wang, H. X., Rosebrock, A. P., & Konopka, J. B. (2010). Identification of GIG1, a GlcNAc-induced gene in Candida albicans needed for normal sensitivity to the chitin synthase inhibitor nikkomycin Z. *Eukaryotic cell*, *9*(10), 1476-1483.
11. Hewald, S., Josephs, K., & Bölker, M. (2005). Genetic analysis of biosurfactant production in Ustilago maydis. *Appl. Environ. Microbiol.*, *71*(6), 3033-3040.
12. Hewald, S., Linne, U., Scherer, M., Marahiel, M. A., Kämper, J., & Bölker, M. (2006). Identification of a gene cluster for biosynthesis of mannosylerythritol lipids in the basidiomycetous fungus Ustilago maydis. *Appl. Environ. Microbiol.*, *72*(8), 5469-5477.
13. Lanver, D., Berndt, P., Tollot, M., Naik, V., Vranes, M., Warmann, T., & Kahmann, R. (2014). Plant surface cues prime Ustilago maydis for biotrophic development. *PLoS pathogens*, *10*(7).
14. Lubkowitz, M. A., Hauser, L., Breslav, M., Naider, F., & Becker, J. M. (1997). An oligopeptide transport gene from Candida albicans. *Microbiology*, *143*(2), 387-396.
15. Ma, J., Dobry, C. J., Krysan, D. J., & Kumar, A. (2008). Unconventional genomic architecture in the budding yeast Saccharomyces cerevisiae masks the nested antisense gene NAG1. *Eukaryotic cell*, *7*(8), 1289-1298.
16. Mueller, A. N., Ziemann, S., Treitschke, S., Aßmann, D., & Doehlemann, G. (2013). Compatibility in the Ustilago maydis–maize interaction requires inhibition of host cysteine proteases by the fungal effector Pit2. *PLoS pathogens*, *9*(2).
17. Panzer, S., Brych, A., Batschauer, A., & Terpitz, U. (2019). Opsin 1 and Opsin 2 of the Corn Smut Fungus Ustilago maydis Are Green Light-Driven Proton Pumps. *Frontiers in microbiology*, *10*, 735.
18. Park, H. S., Lee, S. C., Cardenas, M. E., & Heitman, J. (2019). Calcium-Calmodulin-Calcineurin Signaling: A Globally Conserved Virulence Cascade in Eukaryotic Microbial Pathogens. *Cell host & microbe*, *26*(4), 453-462.Rzechonek, D. A., Day, A. M., Quinn, J., & Mirończuk, A. M. (2018). Influence of ylHog1 MAPK kinase on Yarrowia lipolytica stress response and erythritol production. *Scientific reports*, *8*(1), 14735.
19. Rappas, M., Niwa, H., & Zhang, X. (2004). Mechanisms of ATPases-A multi-disciplinary approach. *Current Protein and Peptide Science*, *5*(2), 89-105.
20. Saika, A., Koike, H., Fukuoka, T., Yamamoto, S., Kishimoto, T., & Morita, T. (2016). A gene cluster for biosynthesis of mannosylerythritol lipids consisted of 4-O-β-D-mannopyranosyl-(2R, 3S)-erythritol as the sugar moiety in a basidiomycetous yeast Pseudozyma tsukubaensis. *PloS one*, *11*(6).
21. Samanta, S. K., & Ghosh, S. K. (2012). The chitin biosynthesis pathway in Entamoeba and the role of glucosamine-6-P isomerase by RNA interference. *Molecular and biochemical parasitology*, *186*(1), 60-68.
22. Sánchez-Arreguin, J. A., Cabrera-Ponce, J. L., León-Ramírez, C. G., Camargo-Escalante, M. O., & Ruiz-Herrera, J. (2019). Analysis of the photoreceptors involved in the light-depending basidiocarp formation in Ustilago maydis. *Archives of microbiology*, 1-11.
23. Segall, L., Javaid, Z. Z., Carl, S. L., Lane, L. K., & Blostein, R. (2003). Structural basis for α1 versus α2 isoform-distinct behavior of the Na, K-ATPase. *Journal of Biological Chemistry*, *278*(11), 9027-9034.
24. Winterberg, B., Uhlmann, S., Linne, U., Lessing, F., Marahiel, M. A., Eichhorn, H., & Schirawski, J. (2010). Elucidation of the complete ferrichrome A biosynthetic pathway in Ustilago maydis. *Molecular microbiology*, *75*(5), 1260-1271.
