# Supplementary material for: Acid pH Strategy Adaptation through NRG1 in Ustilago maydis
Source: J Fungi (Basel). 2021 Jan 28;7(2):91. doi: 10.3390/jof7020091 (PMC7912220; doi:10.3390/jof7020091)
Supplement: Supplementary file 1 [file jof-07-00091-s001.zip › Supplementary files/Table S6 NRG motifs.docx]

**Supplementary Table 6.** Genes used to generate de model of action of NRG1 and the number of motifs founded in its promoter region.

| ID gen | Name | NRG1 motifs | Function |
| --- | --- | --- | --- |
| UMAG_02244 | NA | - | Protein kinase activity |
| UMAG_11957 | NA | 1 | Phosphorelay sensor kinase activity |
| UMAG_03180 | Wco1 | 4 | Blue light receptor |
| UMAG_02357 | Hog1 | 1 | Mitogen activated protein kinase involved in signal transduction pathway that is activated by changes in the osmolality of the extracellular environment |
| UMAG_10368 | HSF | 3 | Heat shock factor a transcriptional activator of heat shock genes |
| UMAG_03296 | YAP1 | 6 | Activator protein, DNA binding |
| UMAG_10974 | Tetrahydrofolyl polyglutamate synthase | 2 | Catalyzes conversion of folates to polyglutamate derivatives |
| UMAG_04106 | o-methyltranferase B | 3 | Enzymes that use S-adenosyl-L-methionine as a substrate for methyl transfer, creating the product S-adenosyl-L-homocysteine |
| UMAG_12026 | Sam-dependent Methyltransferase | 1 | Methyl transferase |
| UMAG_10476 | Trichodiene synthase | 3 | Lyase enzyme, specifically those carbon-oxygen lyases acting on phosphates |
| UMAG_02387 | Oligopeptide transporter | 1 | Oligopeptide transporter |
| UMAG_11365 | GAL4 | 3 | Transcription factor |
| UMAG_02170 | Lysine methyl transferase | 2 | Protein lysine N-methyltransferase activity |
| UMAG_10426 | Rim101/pacC | 7 | Transcription factor that mediates regulation of both acid- and alkaline-expressed genes in response to ambient pH. |

NA: Not assigned
