# Supplementary figures and images for: Acid pH Strategy Adaptation through NRG1 in Ustilago maydis
Source: J Fungi (Basel). 2021 Jan 28;7(2):91. doi: 10.3390/jof7020091 (PMC7912220; doi:10.3390/jof7020091)

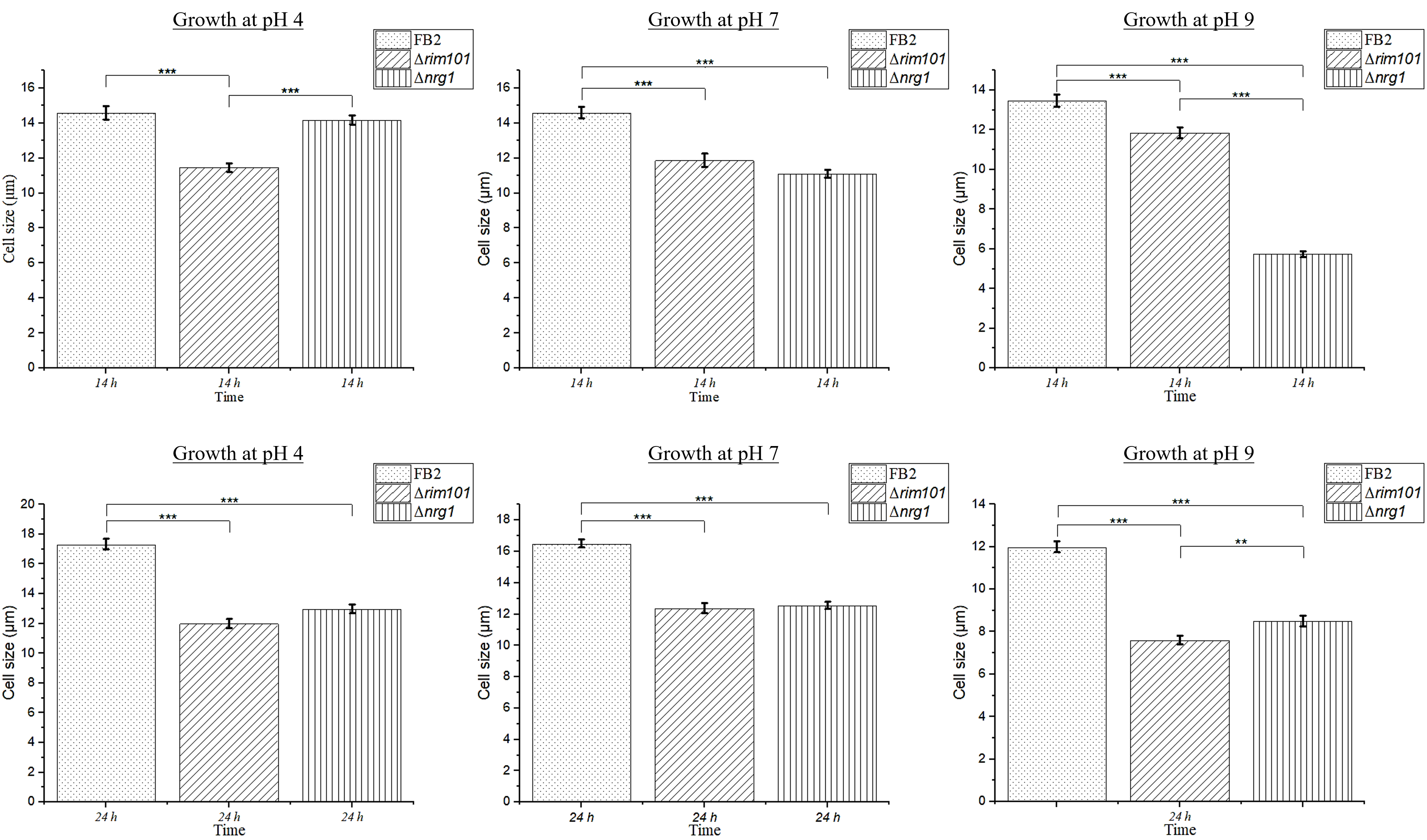

Supplement: Supplementary file 1 [file jof-07-00091-s001.zip › Supplementary files/Fig S1.png]

## Slide 1
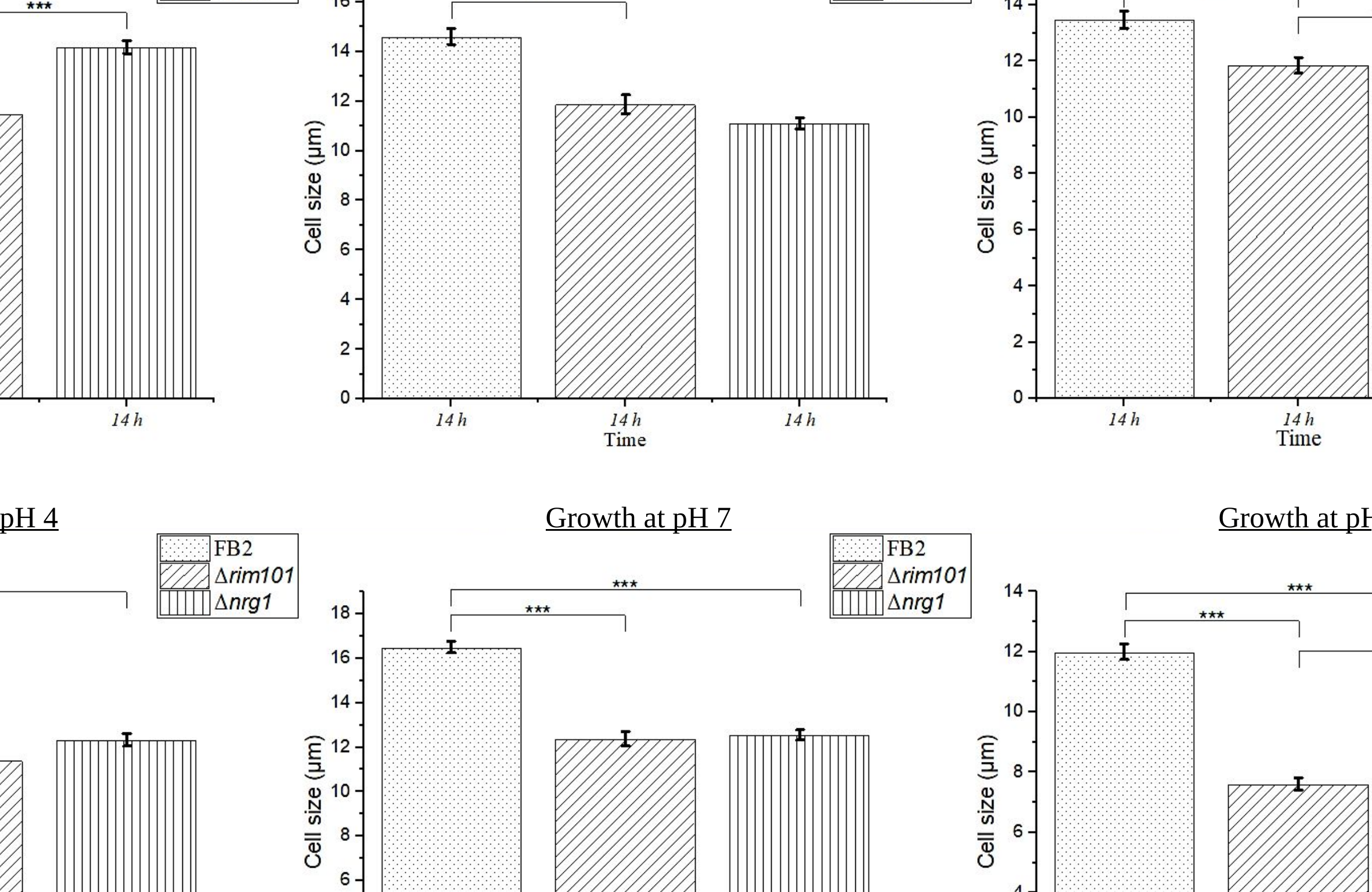

| Growth at pH 4 | Growth at pH 7 | Growth at pH 9 |
| --- | --- | --- |
| Growth at pH 4 | Growth at pH 7 | Growth at pH 9 |
| --- | --- | --- |

Supplement: Supplementary file 1 [file jof-07-00091-s001.zip › Supplementary files/Figure S1 Wild type and mutant strain cell size.pptx]
